# Investigation of Efficacy Enhancing and Toxicity Reducing Mechanism of Combination of Aconiti Lateralis Radix Praeparata and Paeoniae Radix Alba in Adjuvant-Induced Arthritis Rats by Metabolomics

**DOI:** 10.1155/2019/9864841

**Published:** 2019-03-18

**Authors:** Yun-fei Xie, Wu-wen Feng, Mei-chen Liu, Jun Xie, Lei Yu, Xiao-hong Gong, Yun-xia Li, Cheng Peng

**Affiliations:** ^1^School of Pharmacy, State Key Laboratory Breeding Base of Systematic Research, Development and Utilization of Chinese Medicine Resources, Chengdu University of Traditional Chinese Medicine, Chengdu 611137, China; ^2^Department of Nuclear Medicine, Sichuan Academy of Medical Science and Sichuan Provincial People's Hospital, Chengdu 610072, China

## Abstract

Combination of Aconiti Lateralis Radix Praeparata (FZ) and Paeoniae Radix Alba (BS) shows a significant effect in rheumatoid arthritis (RA). This study aimed to investigate the efficacy enhancing and toxicity reducing mechanism of combination of them in adjuvant-induced arthritis (AIA) rats by metabolomics. Rats were randomly divided into seven groups, including A (healthy control), B (model control), C1 (therapy group), C2 (efficacy enhancing group), D1 (toxicity group), and D2 (toxicity reducing group), and dexamethasone group was used as positive control. The plasma biochemical indexes showed that therapeutic dose of lipid-soluble alkaloids of FZ could significantly inhibit the concentrations of IL-1*β*, TNF-*α,* and IFN-*γ* in AIA rats, and combination with total glucosides of peony could further reduce the concentration of IL-1*β*. Then, UPLC-LTQ/Orbitrap MS with untargeted metabolomics was performed to identify the possible metabolites and pathways. Through multivariate data analysis of therapeutic dose groups (A vs. B vs. C1 vs. C2) and multivariate data analysis of toxic dose groups (A vs. B vs. D1 vs. D2), 10 and 7 biomarkers were identified based on biomarker analysis, respectively. After inducing AIA model, the plasma contents of spermidine, vanillylmandelic acid, catechol, and linoleate were increased significantly, and the contents of citric acid, L-tyrosine, L-phenylalanine, leucine, L-tryptophan, and uridine 5'-monophosphate (UMP) were decreased significantly. High dose of lipid-soluble alkaloids of FZ could increase the plasma contents of L-lysine, L-arginine, and deoxycholic acid, while the plasma contents of UMP, carnitine, N-formylanthranilic acid, and adenosine were decreased significantly. The pathway analysis indicated that therapeutic dose of lipid-soluble alkaloids of FZ could regulate energy and amino acid metabolic disorders in AIA rats. However, toxic dose could cause bile acid, fat, amino acid, and energy metabolic disorders. And combination with total glucosides of peony could enhance the therapeutic effects and attenuate the toxicity induced by lipid-soluble alkaloids of FZ.

## 1. Introduction

Rheumatoid arthritis (RA) is an unknown etiology, chronic and inflammatory synovitis-based autoimmune systemic disease, which is characterized by invasive joint inflammation and symmetric lesions of the hands and foot facet joints, accompanied by positive serum anticyclic citrullinated peptide antibody (Anti-CCP) and rheumatoid factor (RF), even involving extra-articular organs and leading to joint deformity and functional injury [1, 2].

In recent years, drugs for the treatment of RA have been continuously put into clinical treatment, such as glucocorticoids, tumor necrosis factor-alpha (TNF-*α*) inhibitors, technetium [99Tc] methylene diphosphonate injection, nonsteroidal anti-inflammatory drugs (NSAIDs), biological targeting agents and disease-modifying antirheumatic drugs (DMARDs). However, they also bring many serious side effects during the treatment of RA, such as Cushing's syndrome, diabetes, hepatotoxicity, and even malignant tumors [3]. Therefore, it is urgently needed to find some drugs with effective and less side effects to solve clinical needs.

Aconiti Lateralis Radix Praeparata (Fuzi in Chinese, FZ), the daughter root of* Aconitum carmichaelii *Debx., has been used for more than a thousand years in clinical practice. In the documents of many classical Chinese medicine books, FZ can treat a variety of diseases, such as polyarthralgia, syncope, acute rheumatic fever, vomiting and diarrhea of asthenia cold, rheumatoid arthritis, heart failure, and inflammation [4–6]. Some studies reported that Aconitum alkaloids were the main bioactive components of FZ, which mainly consist of monoester-diterpenoid aconitines (MDAs) and diester-diterpenoid aconitines (DDAs). However, they were also reported to induce toxic reactions such as gastrointestinal toxicity, neurotoxicity, and fatal cardiac toxicity [7, 8]; therefore, excessive use of FZ in the treatment of RA might have serious consequences.

Paeoniae Radix Alba (Baishao in Chinese, BS), the dry root of* Paeonia lactiflora* Pall., has been widely used in the treatment of many diseases. According to the theory of traditional Chinese medicine, BS has good clinical efficacy in the treatment of menstrual irregularities, blood deficiency, sallow complexion, hypochondriac pain, spontaneous sweating, and headache [4]. In recent years, many studies showed that BS has obvious pharmacological effects, such as anti-inflammatory, antivirus, treating headache and vertigo, spasmolysis, pain relief, irregular menstruation, and liver protection [9, 10]. Total glucosides of peony were the main bioactive components of BS, which mainly consist of paeoniflorin, peony glucoside, hydroxy-paeoniflorin, benzoylpaeoniflorin, and albiflorin. Among them, paeoniflorin accounts for about 90% of total glucosides of peony [11].

Different from western medical system, traditional Chinese medicine focuses more on “system-to-system” treatment mode and pays more attention to pathological and physiological changes [12]. Combinatorial intervention is the main form of traditional Chinese medicine medication, which is summarized from long-term clinical experience. Through the appropriate combination of drugs, it can play an important role in enhancing efficacy and reducing toxicity in clinical treatment. Combinatorial intervention of FZ and BS is the most extensive used prescription in the treatment of RA; the commonly used combination proportions include 2:1, 1:1, and 1:2. Previous studies showed that after combination, the content of ester alkaloids decreased; therefore, the toxicity of Aconitum plants also decreased [13]. In the process of clinical treatment, although FZ is effective in the treatment of RA, excessive use of it will bring toxicity and side effects. Combination of FZ and BS can attenuate toxicity and increase efficiency; however, the mechanism is still unclear and needs to be studied.

Currently, metabolomics is a very popular research in the pharmaceutical field. It focuses on the study of the category, quantity, and variation of organism endogenous metabolites. Through a systematic and comprehensive research of the biochemical content change of cells, tissues or organism, metabolomics can detect and quantify the metabolic fingerprint differences in samples. Combined with pattern recognition method and other chemical analysis techniques, metabolomics can obtain the corresponding biomarkers. Metabolomics is capable of investigating the relationship between pathological and physiological changes by qualitative and quantitative analysis of potential biomarkers [14]. Metabolomics is suitable for the complex system study such as traditional Chinese medicine in order to explore its scientific connotation.

Through our study, the plasma metabolomics based on the UPLC-LTQ/Orbitrap MS technique combined with pattern recognition method, was applied to explore the endogenous metabolites changes caused by combination of lipid-soluble alkaloids of FZ and total glucosides of peony in adjuvant-induced arthritis (AIA) rats. Through the analysis of metabolite pathway changes, the efficacy enhancing and toxicity reducing mechanism of combination of two traditional Chinese medicines in the treatment of RA was investigated. This study can provide a scientific basis for rational use of the two Chinese herbal medicines.

## 2. Materials and Methods

### 2.1. Chemicals and Materials

Acetonitrile, methanol, and formic acid were HPLC grade and obtained from Merck Serono Co., Ltd. (Darmstadt, Germany), Shanghai URChem Co., Ltd. (Shanghai, China), and TCI development Co., Ltd. (Tokyo, Japan), respectively. Purified water was prepared by Arium mini ultrapure water system (Sartorius, Münster, Germany). Rat IL-1*β*, TNF-*α,* and IFN-*γ* ELLSA kits were obtained from Multi Sciences Co., Ltd. (Hangzhou, China). Complete Freund's adjuvant (CFA) was obtained from Sigma-Aldrich Co., Ltd. (St. Louis, MO, USA). Dexamethasone acetate tablets were purchased from Chongqing Kerui Pharmaceutical (group) Co., Ltd. (Chongqing, China). Aconiti Lateralis Radix Praeparata (FZ) was purchased from Sichuan Jiangyou Zhongba Fuzi Technology Development Co., Ltd. (Jiangyou, China). Paeoniae Radix Alba (BS) was purchased from Anhui Bozhou herbal medicine market (Bozhou, China). Professor Jin Pei of Chengdu University of Traditional Chinese Medicine identified the medicinal materials (Chengdu, China).

### 2.2. Drugs Preparation

The extracts of lipid-soluble alkaloids of FZ and total glucosides of peony were extracted from FZ and BS, respectively. 400 g FZ yielded 1 g lipid-soluble alkaloids of FZ, and 25 g BS yielded 1 g total glucosides of peony. The contents of aconitine, mesaconitine, and hypaconitine in lipid-soluble alkaloids of FZ were 0.264 % (2.64 mg/g), 8.24 % (82.4 mg/g), and 1.08 % (10.8 mg/g), respectively. The content of paeoniflorin in total glucosides of peony was 81.6 % (816 mg/g).

### 2.3. Animals

70 male SPF grade Sprague-Dawley rats (180 ± 20 g) were commercially purchased from Laboratory Animal Center of Sichuan Provincial Academy of Chinese Medical Sciences. The number of certification was SCXK 2013-19. All rats were acclimated for a week before the experiments began. They were placed in an appropriate environment, with 12 h light and dark cycle, average temperature: at 20 ± 2°C, and relative humidity: at 55 ± 5 %. The rats were provided with distilled water and rat food. The ethical review committee of Chengdu University of Traditional Chinese Medicine approved all experimental methods and procedures of this study.

### 2.4. AIA Rats Construction and Treatment

SD rats were randomly divided into seven groups, with 10 rats in each group (2 rats in D2 group died due to mistakes in the operation of the intragastric administration). Namely healthy control group (A), AIA model group (B), therapy group (C1), efficacy enhancing combination group (C2), toxicity group (D1), toxicity reducing combination group (D2) and dexamethasone group (DG). The rats in groups B, C1, C2, D1, D2, and DG were injected 0.1 mL CFA in the right hind foot pad, and the rats in group A were injected 0.1 mL 0.9 % sodium chloride injection [15].

19 days after the immunization, all groups began treatment through intragastric administration. Groups A and B were given distilled water as controls, the rats in C1 group received about one-third of the maximum tolerated dose of lipid-soluble alkaloids of FZ as a therapeutic dose (12.5 mg·kg^−1^·d^−1^), the rats in C2 group received lipid-soluble alkaloids of FZ (12.5 mg·kg^−1^·d^−1^) and total glucosides of peony (400 mg·kg^−1^·d^−1^), the rats in D1 group received a maximum tolerated dose of lipid-soluble alkaloids of FZ (35 mg·kg^−1^·d^−1^), the rats in D2 group received lipid-soluble alkaloids of FZ (35 mg·kg^−1^·d^−1^) and total glucosides of peony (1120 mg·kg^−1^·d^−1^), and the rats in DG group received dexamethasone (0.3 mg·kg^−1^·d^−1^).

### 2.5. Pharmacological Effects Evaluation

After the immunization, rats were weighed every 3 days. In order to record the development of arthritis erythema or edema in rat paws and observe the effects of the drugs, the paw volumes were measured every 4 days and the arthritis scores were evaluated simultaneously. The semiquantitative clinical score was used to assess the severity of arthritis: 0, normal joint condition; 1, slight swelling and erythema of the finger or ankle; 2, moderate swelling and erythema of the ankle; 3, swelling and erythema of the whole rat paw including fingers; and 4, severe arthritis of the limb and involvement of multiple joints. The recorded paw volumes were the average volumes of the two hind paws, and the recorded arthritis scores were the sum of the two hind paws [16].

### 2.6. Sample Collection

Drug administration lasted for 14 days. Two hours after last administration, all rats were anesthetized with 10 % chloral hydrate, 0.35 mL/100 g per rat. The blood samples were collected into EDTA tube from the rat abdominal aorta then immediately centrifuged at 1,600 g for 15 min at 4°C. The plasma samples were transferred into sterilized cryopreservation tube for metabolomics analysis and ELISA examinations. The plasma samples were stored at -80°C and thawed at 4°C before analysis.

### 2.7. ELISA Examinations

The levels of cytokines IL-1*β*, TNF-*α,* and IFN-*γ* in rat plasma were evaluated by ELISA kits.

### 2.8. Metabolomics Analysis Based on UPLC-LTQ/Orbitrap MS

UPLC-LTQ/Orbitrap MS analysis was based on Waters ACQUITY UPLC system combined with electrospray ionization (ESI) source and Thermo LTQ/Orbitrap XL mass spectrometry. The analysis equipment had an autosampler and a multisolvent delivery system. Analysis was carried out on an ACQUITY UPLC BEH C_18_ column (100×2.1 mm, 1.7 *μ*m, Waters). The column and autosampler temperature were maintained at 40°C and 4°C, respectively. Solvent A (water contained 0.1% formic acid) was mixed with solvent B (acetonitrile contained 0.1% formic acid) in different proportions for gradient elution of analytes, and the flow rate was set at 0.25 mL/min. After equilibration, 5 *μ*L supernatant plasma of each sample were injected into UPLC-LTQ/Orbitrap MS system for metabolomics analysis. Solvent B with an increasing linear gradient was used as follows: 0~1 min, 2% B; 1~9.5 min, 2%~50% B; 9.5~14 min, 50%~98% B; 14~15 min, 98% B; 15~15.5 min, 98%~2% B; 15.5~17 min, 2% B.

MS condition: the Thermo LTQ/Orbitrap XL mass spectrometer was applied to ESI-MS experiments. The spray voltages in positive and negative ion modes were 4.8 kV and 4.5 kV, respectively. Sheath and auxiliary gas were set at 45 and 15 arbitrary units, respectively. The normalized collision energy was set at 30 eV. The capillary temperature was maintained at 325°C. The capillary voltages in positive and negative modes were 35 V and -15 V, and the tube lens voltages in positive and negative modes were 50 V and -50 V.

### 2.9. Data Analysis

The Protein Wizard software was used to convert the analysis data of UPLC-LTQ/Orbitrap MS into CDF format. And then, XCMS (www.bioconductor.org/) was applied to preprocessing of the study. Peak areas of metabolites in each sample were normalized by sum method from metaboanalyst (www.metaboanalyst.ca). The data sets from all samples, including normalized peak area of metabolites and retention time, were used for data preprocessing by Simca-P software, version 13.0 (Umetrics AB, Umea, Sweden). The unit variance scaling and mean-centered method were applied to data processing. And then, principal component analysis (PCA) and partial least squares-discriminant analysis (PLS-DA) were used to pattern recognition analysis by multivariate statistical analysis tool. In PLS-DA model, variable importance in the projection (VIP) value higher than 1 was considered a potential variable. The SPSS software, version 21.0 (Chicago, IL, USA), was used for the analysis of potential variables by Student's* t*-test and one-way analysis of variance (ANOVA), and* P* < 0.05 was considered to be statistically different.

The potential metabolic biomarkers were identified by MS/MS fragmentation patterns and matching the exact molecular weight to the online database (the mass error was less than 20 ppm), mainly including HMDB database (http://www.hmdb.ca), Metlin database (https://metlin.scripps.edu), Massbank database (http://massbank.eu/MassBank), Lipid Maps database (http://www.lipidmaps.org), and Mzcloud database (https://www.mzcloud.org). Then KEGG database (http://www.kegg.jp) was applied to find out the correlation between candidate biomarkers and plot metabolomics pathways network diagram.

## 3. Results

### 3.1. Evaluation of AIA Model and Therapeutic Effects of Drugs

**Figure 1:**
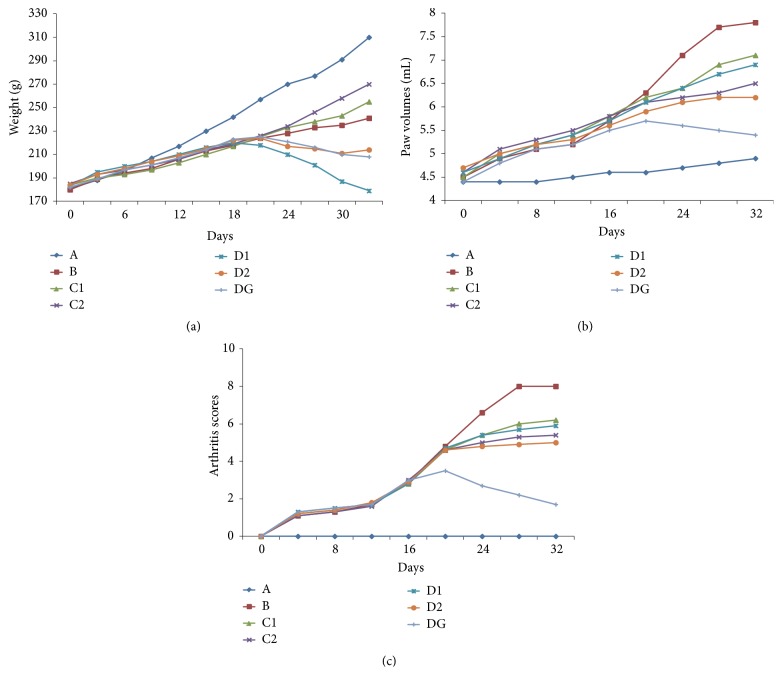
(a) The body weight of rats in each group after immunization, weighed every 3 days. (b) The change of paw volumes of rats in each group after immunization, measured every 4 days. (c) The clinical arthritis scores in each group after immunization, evaluated every 4 days. Healthy control group (A). AIA model group (B). Therapy group (C1). Efficacy enhancing combination group (C2). Toxicity group (D1). Toxicity reducing combination group (D2). Dexamethasone group (DG).

After immunization with CFA, swelling immediately appeared on the injected feet, on the 8th day after immunization, swelling and erythema gradually appeared on the left foot, and the model group all reached the peak of inflammation on the 28th day. As shown in Figure 1(a), C1, C2 groups had higher body weight than model group; however, the body weight of D1, D2, and dexamethasone groups were significantly reduced after administration. As shown in Figures 1(b) and 1(c), the paws swelling and arthritis scores in each group were significantly increased after immunization; however, in drug administration groups, the paws swelling and arthritis scores were significantly inhibited after administration (*P* < 0.05 vs. model group). The above results illustrated that dexamethasone had a strong anti-inflammatory effect, lipid-soluble alkaloids of FZ also had a good effect on AIA rats, and combination with total glucosides of peony could enhance this effect. However, excessive lipid-soluble alkaloids of FZ could cause toxic damage to rats.

### 3.2. Plasma Biochemical Parameters

Inflammatory cytokines, such as IL-1*β* and TNF-*α*, played an important role in the inflammatory response of RA. They could act on synoviocytes, endothelial cells, bone-derived cells, and chondrocytes to produce collagenase and synthesize PGE_2_ in articular lesions. Especially, IL-1*β* was the main cause of bone and cartilage destruction [17]. IFN-*γ* was an inflammatory cytokine with the functions of immune system regulation and cell proliferation, and it was also considered to be an important marker of RA [18]. The ELISA results were shown in Table 1. The levels of IL-1*β*, TNF-*α,* and IFN-*γ* in model group were significantly higher than control group (*P* < 0.05). After administration, the levels of IL-1*β*, TNF-*α,* and IFN-*γ* were significantly reduced in dexamethasone group, the levels of IL-1*β* were significantly reduced in C1 group (*P* < 0.05), C2 group (*P* < 0.001), D1 group (*P* < 0.05), and D2 group (*P* < 0.001), the levels of TNF-*α* were also reduced in all administration groups, and the levels of IFN-*γ* were significantly reduced in C1 group (*P* < 0.01), C2 group (*P* < 0.01), D1 group (*P* < 0.05), and D2 group (*P* < 0.01). The results indicated that lipid-soluble alkaloids of FZ had anti-inflammatory effects on the inhibition of IL-1*β*, TNF-*α,* and IFN-*γ*, and combination with total glucosides of peony could enhance the regulation of IL-1*β*. However, beyond a certain range, the levels of IL-1*β*, TNF-*α,* and IFN-*γ* were not reduced continually when the dose of lipid-soluble alkaloids of FZ increased; instead, toxic reactions occurred.

### 3.3. Multivariate Analysis of UPLC-LTQ/Orbitrap MS Data

Plasma chromatography in positive and negative ion modes was obtained by UPLC-LTQ/Orbitrap MS. The plasma metabolites of each sample were investigated by multivariate statistical analysis, to obtain the difference of metabolic components.

**Figure 2:**
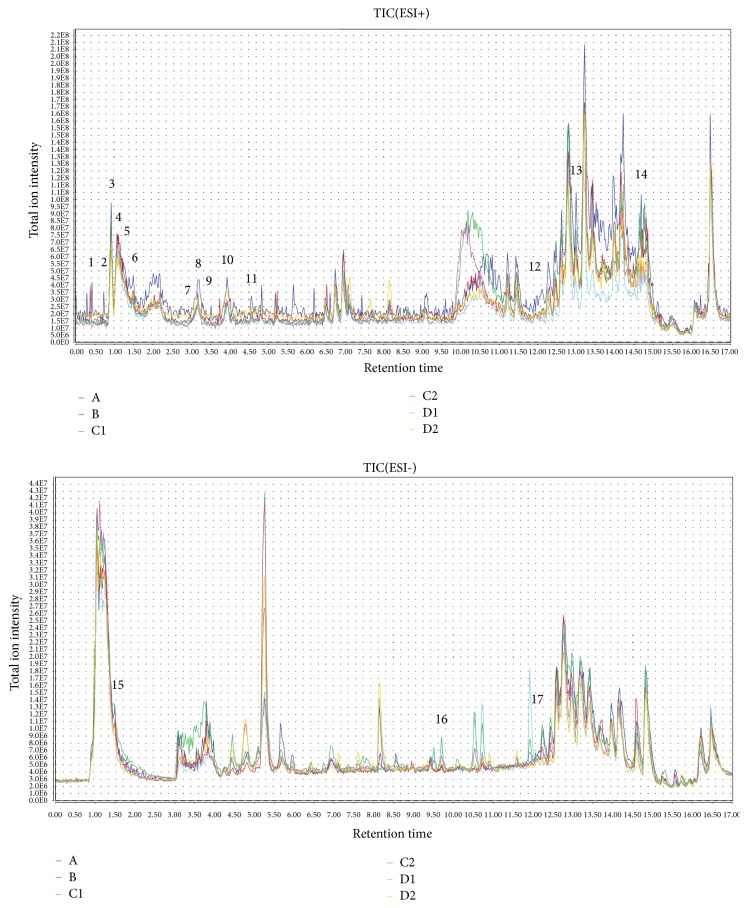
UPLC-LTQ/Orbitrap MS TIC chromatograms of the plasma samples from six groups in positive and negative modes. 1. Uridine 5'-monophosphate (UMP); 2. Spermidine; 3. L-Lysine; 4. Carnitine; 5. L-Arginine; 6. L-Tyrosine; 7. N-Formylanthranilic acid; 8. L-Phenylalanine; 9. Leucine; 10. L-Tryptophan; 11. L-Isoleucine; 12. Vanillylmandelic acid; 13. Catechol; 14. Linoleate; 15. Citric acid; 16. Adenosine; 17. Deoxycholic acid. Healthy control group (A). AIA model group (B). Therapy group (C1). Efficacy enhancing combination group (C2). Toxicity group (D1). Toxicity reducing combination group (D2).

Through the conditions listed in Section 2.8, UPLC-LTQ/Orbitrap MS analysis could detected 1632 precursor molecules in positive ion mode and 3137 precursor molecules in negative ion mode, within 17 min. Typical total ion chromatograms (TICs) of UPLC-LTQ/Orbitrap MS in positive and negative modes were shown in Figure 2, some differences between each group could be observed by TICs. A total of 17 potential biomarkers associated with therapy and toxicity mechanism were marked in TICs. It could be observed that the screened potential biomarkers had obvious signal intensity differences in each group.

In order to clarify the relationship between therapeutic dose and toxic dose of lipid-soluble alkaloids of FZ, the efficacy enhancing and toxicity reducing mechanism after combination with total glucosides of paeony, multivariate data analysis was carried out in two comparison groups, namely, multivariate data analysis of therapeutic dose groups (A vs. B vs. C1 vs. C2) and multivariate data analysis of toxic dose groups (A vs. B vs. D1 vs. D2). After peak matching, all variables of each group were analyzed by PCA. In the clustering of the data, the abnormal samples were removed. The PCA score plots in positive and negative modes were shown in supplementary materials (Available here).

**Figure 3:**
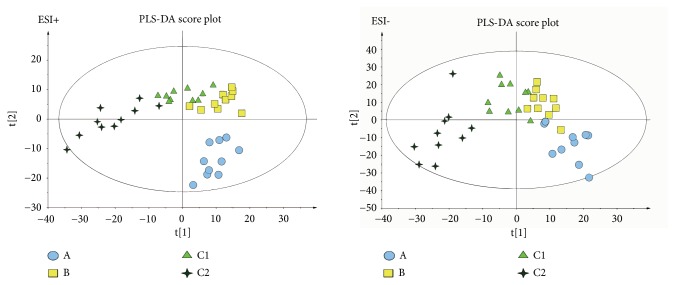
The PLS-DA score plot of therapeutic dose groups (A vs. B vs. C1 vs. C2) in ESI+ and ESI−. Healthy control group (A). AIA model group (B). Therapy group (C1). Efficacy enhancing combination group (C2).

**Figure 4:**
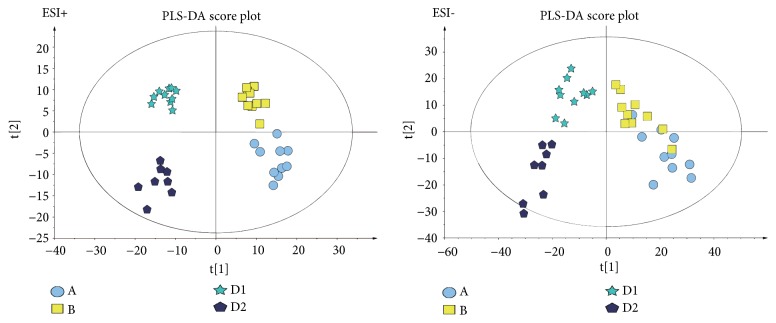
The PLS-DA score plot of toxic dose groups (A vs. B vs. D1 vs. D2) in ESI+ and ESI−. Healthy control group (A). AIA model group (B). Toxicity group (D1). Toxicity reducing combination group (D2).

**Figure 5:**
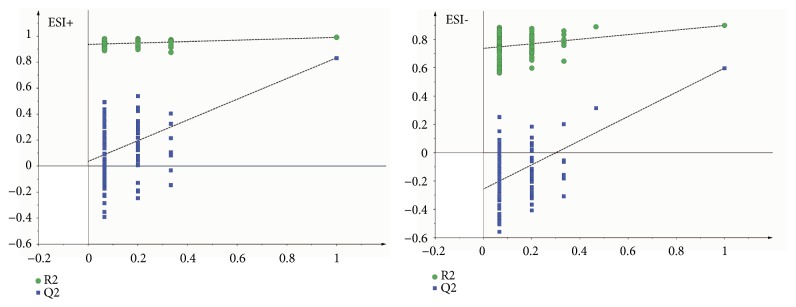
100-permutation test of PLS-DA model of therapeutic dose groups (A vs. B vs. C1 vs. C2) in ESI+ and ESI−.

**Figure 6:**
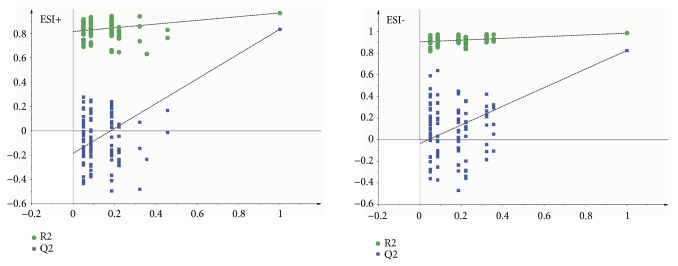
100-permutation test of PLS-DA model of toxic dose groups (A vs. B vs. D1 vs. D2) in ESI+ and ESI−.

**Figure 7:**
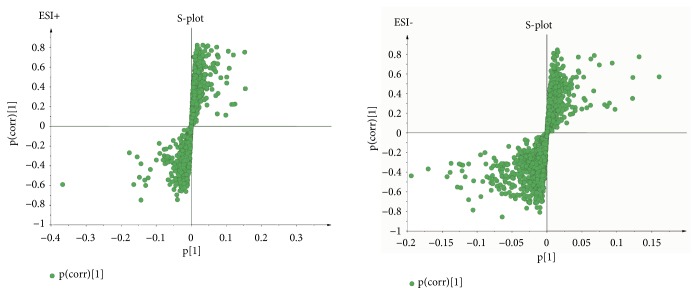
The PLS-DA S-plot in ESI+ and ESI−.

As the supervised analysis method of multivariate analysis, PLS-DA was applied to further distinguish the differences of analysis groups and confirm the potential biomarkers by VIP value. PLS-DA score plots in positive and negative modes were shown in Figures 3 and 4, the PLS-DA parameter of therapeutic dose groups (positive ion mode: R^2^X=0.463, R^2^Y=0.981, Q^2^=0.804; negative ion mode: R^2^X=0.421, R^2^Y=0.922, Q^2^=0.531) and toxic dose groups (positive ion mode: R^2^X=0.380, R^2^Y=0.978, Q^2^=0.850; negative ion mode: R^2^X=0.515, R^2^Y=0.986, Q^2^=0.850). The results of PLS-DA model showed an obvious separation trend among all groups; the samples of each group were separated from each other and clustered together individually. The model group and the control group were separated clearly, which indicated that the animal model was successful, and the occurrence of disease could cause significant changes in endogenous metabolites. Compared with the two low-dose groups, the two high-dose groups were further apart from the model group and the control group and clustered in another area individually, which suggested that the occurrence of toxicity might cause great changes in endogenous metabolites. Permutation tests in Figures 5 and 6 were applied 100 iterations to detect whether the PLS-DA models were overfitting; the results suggested that they were not overfitted. The S-plot was shown in Figure 7, which could visualize the relationship between correlation and covariance of PLS-DA. As shown in the picture, each spot corresponded to an endogenous substance, and the variables situated at the ends of the S-plot contributed highly to the differentiation.

### 3.4. Identification and Analysis of Potential Biomarkers

**Figure 8:**
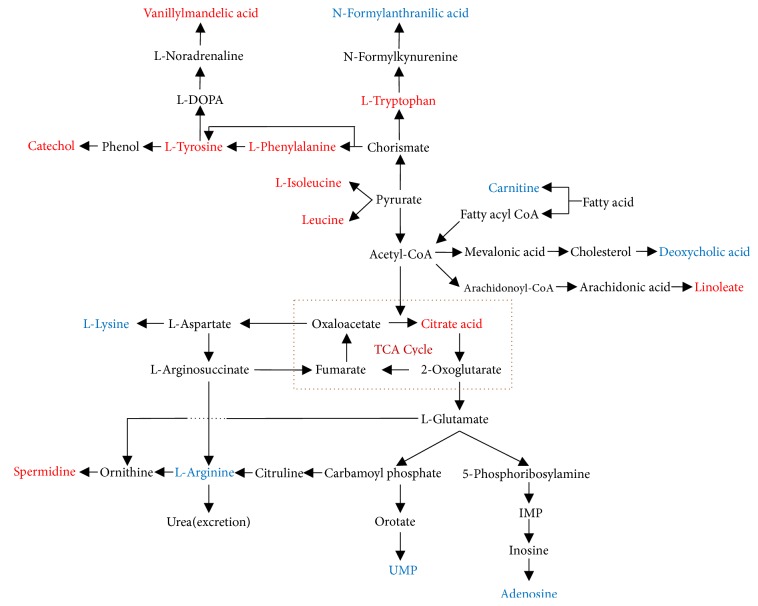
The metabolic pathway networks of potential biomarkers in response to therapeutic and toxic mechanism of combination of FZ and BS in AIA rats. The red marked metabolites were associated with the therapeutic mechanism. The blue marked metabolites were associated with the toxic mechanism.

According to VIP values in PLS-DA model, the potential biomarkers with VIP > 1 were selected for further Student's* t*-test and ANOVA analysis, and* P* < 0.05 was considered to be statistically different. Following these steps and threshold above, differential metabolites related to therapeutic or toxic mechanism of drugs in AIA rats were screened out. Based on the retention time, exact mass and MS/MS fragmentation patterns information, the ions of metabolites were identified by standard sample spectra and online database information. The mass spectra of the identified compounds were shown in supplementary materials. As listed in Table 2, 10 metabolic biomarkers were considered as potential biomarkers related to therapy mechanism of lipid-soluble alkaloids of FZ and total glucosides of peony. As listed in Table 3, 7 metabolic biomarkers were considered as potential biomarkers related to toxicity mechanism of lipid-soluble alkaloids of FZ, and toxicity reducing mechanism of combination with total glucosides of peony. The identified metabolic biomarkers referenced to the KEGG database (http://www.kegg.jp) in order to investigate the interrelationship of the potential metabolic pathways involved in the development of RA, and the therapeutic or toxic mechanism of the two Chinese herbal medicines in the treatment of RA [19]. The metabolic pathway networks of potential biomarkers were shown in Figure 8.

## 4. Discussion 

### 4.1. Analysis of Metabolites Associated with Therapeutic Effects

Nontargeted metabolomics was applied to analyze the therapeutic mechanism of lipid-soluble alkaloids of FZ and total glucosides of peony. The potential biomarkers were screened out by multivariate data analysis of therapeutic dose groups (A vs. B vs. C1 vs. C2), and the results showed that the therapeutic mechanism was related to the regulation of energy and amino acids metabolism.

#### 4.1.1. Metabolites Alteration Related to Energy Metabolism

In the process of many diseases, with the occurrence and development of inflammation, the consumption of energy in the body would be reflected in the metabolites.

Citric acid was an important intermediate in mitochondria to participate tricarboxylic acid (TCA) cycle. TCA cycle was ubiquitous metabolic pathway in aerobic organisms and played an important role in energy metabolism. The levels of citric acid in urine and plasma of patients with bone diseases decreased significantly than those of normal people [20]. In our study, the level of citric acid was significantly decreased in model group, which suggested that the citric acid synthase in TCA cycle was impaired in AIA rats. Compared with model group, the levels of citric acid in C1 and C2 groups were significantly upregulated, especially in C2 group, which indicated that lipid-soluble alkaloids of FZ might regulate TCA cycle by increasing citric acid level, and total glucosides of peony could enhance this effect.

Linoleate was an essential polyunsaturated fatty acid, widely distributed in the nutrients needed by the human body. Linoleate was also a precursor of arachidonic acid and involved in the biosynthesis of many eicosanoid derivatives. Some studies clearly demonstrated that linoleate had strong proinflammatory properties and was directly associated with metabolic diseases [21]. As the epoxides of arachidonic acid and linoleate, epoxyeicosatrienoic acids (EETs) and epoxyoctadecenoic acids (EpOMEs) could be converted to dihydroxyeicosatrienoic acids (DHETEs) and dihydroxyoctadecenoic acids (DHOMEs), two compounds possessing mild anti-inflammatory and proinflammatory properties, respectively [22–24]. Furthermore, 9-HODE was another metabolite of linoleate, which could promote a strong proinflammatory reaction in wound-healing model rats [25]. In our study, the level of linoleate was significantly increased in model group, while the levels of linoleate in C1 and C2 groups were lower than that in model group, especially in C1 group, which suggested that lipid-soluble alkaloids of FZ might reduce the level of linoleate and thus lead to an anti-inflammatory response. However, the synergistic effect of combination with total glucosides of peony was not obvious.

#### 4.1.2. Metabolites Alteration Related to Amino Acids Metabolism

Some studies demonstrated that amino acids were the main differential metabolites in plasma of RA patients. Amino acids played an essential role in the pathogenesis of RA. For example, inflammation and synovial hyperplasia were closely related to the alteration of amino acids metabolism* in vivo* [26–28].

L-tryptophan was an essential amino acid, which played an important role in physiological process of the body. Through the kynurenine metabolic pathway, tryptophan metabolism was considered to modulate the balance of immune system between tolerance to nonharmful antigens and responsiveness to pathogens. Interferon-gamma (IFN-*γ*), which is released by activated T-cells and leucocytes, was a proinflammatory cytokine* in vivo*. IFN-*γ* could induce catabolism of tryptophan during many different cell types of immune response [29]. Tryptophan depletion was closely related to the defense mechanism during inflammation by suppressing the proliferation of invading pathogens [16]. The decreased level of L-tryptophan in model group was closely related to immune response of RA, and C2 group could significantly increase the level of L-tryptophan. The above results suggested that combination of lipid-soluble alkaloids of FZ and total glucosides of peony had obvious regulation of tryptophan metabolism pathway.

L-phenylalanine, L-isoleucine, and leucine were all essential amino acids of the human body, and L-phenylalanine was also a precursor of L-tyrosine [30, 31]. The levels of tyrosine, phenylalanine, isoleucine, valine, alanine, and histidine had lower concentrations in patients with rheumatoid arthritis and systemic lupus erythematosus [32, 33]. Previous studies showed that AIA rats had more phenylalanine excretion, which suggested that phenylalanine metabolism could be partially blocked in inflammatory state and the protein metabolism might be disturbed [34, 35]. Leucine assumed a crucial role in the regulation of protein metabolism, the decreased level of leucine in plasma indicated a disorder in the regulation of protein degradation. Leucine could modulate proinflammatory cytokines and increase anti-inflammatory cytokines to maintain immune balance in the body [36]. As the results showed, L-phenylalanine, L-tyrosine, L-isoleucine, and leucine were significantly altered in model group. The changed levels of these four biomarkers indicated that leucine, isoleucine and valine biosynthesis pathway, tyrosine, tryptophan and phenylalanine biosynthesis pathway, and phenylalanine metabolism pathway had been disturbed in AIA rats. Compared with model group, C2 group could upregulate the levels of these amino acids in different degrees, and the effects were better than C1 group. The above results indicated that combination of lipid-soluble alkaloids of FZ and total glucosides of peony were superior to single dose of lipid-soluble alkaloids of FZ in the treatment of RA.

Spermidine was a polyamine with important physiological functions and played a crucial role in cell growth, aging, autophagy, and neurodegenerative diseases. Polyamines levels increased in many diseases, such as Alzheimer's disease, malignant tumor, Parkinson's disease, and inflammatory diseases. Many diseases were associated with inflammation, while polyamines were involved in inflammatory responses, the levels of polyamines increased with the development of inflammation [37]. Previous studies suggested that spermidine and its oxidative metabolites, mediated the anti-inflammatory activity in human rheumatoid synovial fluid, plasma of arthritic rats and inflammatory exudates [38]. Spermidine could protect macrophages from inflammatory injury by increasing the synthesis of anti-inflammatory factor IL-10 and inhibiting the expression of proinflammatory cytokines IL-12 and IFN-*γ* [39]. As the results showed that the level of spermidine was significantly increased in model group. Compared with model group, the level of spermidine decreased significantly in C1 group, but the therapeutic effect of C2 group was not obvious. The above results suggested that lipid-soluble alkaloids of FZ could reduce the level of spermidine to make an anti-inflammatory response. However, the synergistic effect of combination with total glucosides of peony was not significant.

The majority of catechols existed in body as derivatives of norepinephrine, adrenaline, and dopamine; all of them were collectively referred to catecholamines (CAs). And vanillylmandelic acid was the final metabolite of CAs. Catechol and vanillylmandelic acid were all involved in tyrosine metabolism pathway [40, 41]. In addition to regulating some visceral activities such as cardiovascular, respiratory, and digestive activities, CAs was also involved in regulating the immune function of the body [42]. Previous studies showed that CAs could inhibit the production of TNF-*α*, IL-1, and IFN-*γ*, while they inhibit TNF-*α* production by astrocytes, monocytes, and microglial cells and indirectly inhibit IL-1 production by inhibition of TNF-*α* and potentiation of IL-10 production [43]. Some studies also demonstrated the presence of catecholamine-producing cells in the synovial tissue during arthritis but not in normal tissue, which suggested that they were related to chronic inflammation [44]. In this study, the levels of catechol and vanillylmandelic acid were significantly increased in model group. After medication, the levels of catechol in C1 and C2 groups decreased significantly, and the level of vanillylmandelic acid in C2 group decreased significantly. The above results suggested that lipid-soluble alkaloids of FZ had anti-inflammatory effects, and total glucosides of peony could obviously enhance the anti-inflammatory effects in arthritis disease.

### 4.2. Analysis of Metabolites Associated with Toxicity Reduction

Nontargeted metabolomics was applied to analyze the toxicity mechanism of lipid-soluble alkaloids of FZ and the toxicity reducing mechanism of combination with total glucosides of peony. The potential biomarkers were screened out by multivariate data analysis of toxic dose groups (A vs. B vs. D1 vs. D2), and the results showed that the toxicity mechanism was related to the fat, bile acids, amino acids, and energy metabolic disorders.

#### 4.2.1. Metabolites Alteration Related to Fatty Metabolism

As an essential nutrient of the organism, carnitine played an important role in promoting the oxidative decomposition of fatty acids. In the process of fatty metabolism, firstly, in the mitochondrial outer membrane and endoplasmic reticulum, long-chain fatty acid converted to fatty acyl CoA by ester of acyl CoA synthetase [45]. Then, the fatty acyl CoA combined with L-carnitine to form carnityl carnitine by carnitine acyltransferase I (CPT-I), carnityl carnitine transported into mitochondria, and release L-camitine by carnitine acyltransferase II (CPT-II). In mitochondria, fatty acyl CoA underwent *β*-oxidization to synthesize acetyl-CoA [46, 47]. In our study, the levels of carnitine were significantly decreased in D1 group and increased in D2 group. The above results suggested that toxic dose of lipid-soluble alkaloids of FZ could impair the activity of fatty acid esterase or CPT-I due to drug hepatotoxicity, and total glucosides of peony could reduce this toxicity by regulating the metabolic environments of fatty acids.

#### 4.2.2. Metabolites Alteration Related to Bile Acids Metabolism

Deoxycholic acid was a free bile acid, derived from the loss of an oxygen atom of cholic acid which was synthesized by the liver. It served many indispensable physiological functions, such as absorption of vitamins and lipids, and the regulation of glucose and cholesterol [48]. The levels of bile acids were increased in hepatic and intestinal diseases, impaired renal tubules and hepatotoxicity injury, because of the synthesis and clearance of intrahepatic bile acids and their intestinal absorption were disturbed [49]. The damaged liver could lead to obstruction of bile acids enterohepatic circulation, resulting in the increased levels of blood bile acids. Bile acids were also considered as potential biomarkers of drug-induced liver injury [50]. In this study, the levels of deoxycholic acid were significantly increased in D1 and D2 groups, which suggesting that toxic dose of lipid-soluble alkaloids of FZ could cause liver injury, but total glucosides of peony could not prevent this damage.

#### 4.2.3. Metabolites Alteration Related to Amino Acids Metabolism

Amino acids were mainly metabolized in the liver, and any liver injury might cause the disorder of amino acids metabolism. The results in our study showed that the metabolic environments and pathways of amino acids were seriously impacted by toxic dose of lipid-soluble alkaloids of FZ.

N-formylanthranilic acid was the metabolite of tryptophan by kynurenine pathway; then, N-formylanthranilic acid was further converted to quinolinic acid into tryptophan-NAD+ pathway or converted to acetyl-CoA to participate the TCA cycle [51]. The decreased level of N-formylanthranilic acid in D1 group suggested that toxic dose of lipid-soluble alkaloids of FZ might cause tryptophan and NAD metabolic disorders, energy metabolism blocked and hepatic toxicity occurred. However, the regulatory effects of total glucosides of peony on these disorders were not obvious.

L-arginine was usually considered to be a conditionally essential amino acid [52]. L-arginine occupied an important position because of its extensive biological functions; it was the critical intermediate in the urea cycle, the substrate for nitric oxide (NO) synthase, the source of ornithine for polyamine synthesis, and the source of amidino groups for creatine synthesis. In addition, it was also closely related to the metabolism of glutamine and pyrimidine [53, 54]. In liver, L-arginine was hydrolyzed by ornithine cycle, ornithine could subsequently be converted to proline and polyamines, and the production of urea was excreted by the kidney [55]. Other pathways of L-arginine catabolism were synthesis of agmatine, creatine, and NO [56, 57].* In vivo*, pyrimidines synthesized through the formation of carbamoyl phosphate from glutamine, carbamoyl phosphate could also enter the urea cycle to participate the synthesis of L-arginine [58].

Previous studies showed that L-lysine and L-arginine were transported by the same carrier through intestinal mucosal transport, and they were also transported by the same carrier in renal tubule [59]. L-lysine could compete with L-arginine for intracellular transport, excess of L-lysine might indirectly affect L-arginine metabolism [60].

In our study, the levels of L-arginine and L-lysine were significantly increased in D1 group, which suggested that the metabolic disorder of L-arginine in the liver might be induced by lipid-soluble alkaloids of FZ, including suppressed pathways of urea, creatine, creatinine, and ornithine synthesis. Metabolic disorders of L-arginine and L-lysine also interacted with each other because of the same carrier was used by them. The level of uridine 5'-monophosphate (UMP) was downregulated in D1 group, which might also be indirectly caused by the metabolic disorder of L-arginine. The level of UMP was upregulated in D2 group, which indicated that total glucosides of peony could significantly regulate the metabolic disorders caused by hepatotoxicity and maintain the stability of the liver internal environments.

#### 4.2.4. Metabolites Alteration Related to Energy Metabolism

Adenosine was an endogenous nucleoside distributed in human cells and directly involved in the energy metabolism of cells. Adenosine was the material basis for energy storage molecules, nucleic acids, neuromodulator of cellular activity and enzyme's substrate, etc. It was also an essential intermediate for the synthesis of adenylate, adenine and adenosine triphosphate (ATP) [61]. The level of adenosine was significantly decreased in D1 group, and this phenomenon suggested that lipid-soluble alkaloids of FZ could interfere with adenosine metabolism and directly affect the energy metabolism. The level of adenosine was upregulated in D2 group, which indicated that the metabolic disorder of adenosine could be regulated by total glucosides of peony.

## 5. Conclusions

Firstly, through the ELISA experiments, therapeutic dose of lipid-soluble alkaloids of FZ exhibited strong anti-inflammatory activity, it could significantly reduce the concentrations of IL-1*β*, TNF-*α,* and IFN-*γ*, and the combination with total glucosides of peony could further reduce the concentration of IL-1*β*. Then, through metabolomics study, the therapeutic mechanism of combination of lipid-soluble alkaloids of FZ and total glucosides of peony in AIA rats had been investigated. The therapeutic mechanism mainly involved in citrate cycle, phenylalanine, tyrosine, tryptophan metabolism and leucine, isoleucine biosynthesis. The effects of total glucosides of peony in the attenuation of toxicity induced by lipid-soluble alkaloids of FZ were also investigated. The bile secretion, purine, pyrimidine, lysine and arginine metabolic disorders caused by drug-induced liver injury could be regulated by total glucosides of peony.

In summary, this study showed that lipid-soluble alkaloids of FZ were both medicinal and toxic components, and different doses could affect different biological targets and produce different biological effects. Combination with total glucosides of peony could enhance the therapeutic effects and attenuate the toxicity induced by lipid-soluble alkaloids of FZ.

## Figures and Tables

**Table 1:** Effect of combination of FZ and BS on IL-1*β*, TNF-*α,* and IFN-$\gamma \;\;(\overset{-}{X}\pm S)$.

Group	IL-1*β*/pg·mL^−1^	TNF-*α*/pg·mL^−1^	IFN-*γ*/pg·mL^−1^
A group	217.39±61.22	135.20±17.86	154.23±43.04
B group	731.26±156.34^###^	164.24±18.82^#^	506.03±191.55^##^
C1 group	547.62±172.16^*∗*^	145.05±17.85	198.84±54.15^*∗∗*^
C2 group	262.96±191.44^*∗∗∗*^	143.20±18.85	182.98±88.86^*∗∗*^
D1 group	535.62±154.12^*∗*^	146.37±24.72	209.58±69.74^*∗*^
D2 group	375.62±143.54^*∗∗∗*^	148.54±26.84	186.47±71.19^*∗∗*^
Dexamethasone group	239.42±102.37^*∗∗∗*^	140.30±19.10^*∗*^	185.88±67.94^*∗∗*^

Significant differences were based on independent sample *t*-test.

^#^ *P* < 0.05, compared with control group; ^##^*P* < 0.01, compared with control group; ^###^*P* < 0.001, compared with control group.

^*∗*^ *P* < 0.05, compared with model group; ^*∗∗*^*P* < 0.01, compared with model group; ^*∗∗∗*^*P* < 0.001, compared with model group

Healthy control group (A). AIA model group (B). Therapy group (C1). Efficacy enhancing combination group (C2). Toxicity group (D1). Toxicity reducing combination group (D2). Dexamethasone group (DG).

**Table 2:** List of the identification of potential biomarkers among groups.

No.	Biomarker identification	m/z	ppm	ESI mode	Rt/min	Formula	B vs. A	C1 vs. B	C2 vs. B	Pathway
1	Spermidine	146.16467	6.64	+	0.91	C_7_H_19_N_3_	↑^##^	↓^*∗∗*^	↑	Arginine and proline metabolism
2	Citric acid	191.01813	9.03	−	1.52	C_6_H_8_O_7_	↓^#^	↑^*∗*^	↑^*∗∗*^	Citrate cycle
3	L-Tyrosine	182.08048	4.64	+	1.65	C_9_H_11_NO_3_	↓^#^	↓	↑^*∗∗∗*^	Phenylalanine metabolism
4	L-Phenylalanine	166.08602	1.49	+	3.15	C_9_H_11_NO_2_	↓^#^	↑	↑^*∗*^	Phenylalanine metabolism
5	Leucine	132.10130	2.60	+	3.23	C_6_H_13_NO_2_	↓^#^	↑	↑	Valine, leucine and isoleucine biosynthesis
6	L-Tryptophan	205.09676	1.79	+	3.92	C_11_H_12_N_2_O_2_	↓^#^	↑	↑^*∗∗∗*^	Tryptophan metabolism
7	L-Isoleucine	132.10129	2.41	+	4.63	C_6_H_13_NO_2_	↓	↓	↑^*∗*^	Valine, leucine and isoleucine biosynthesis
8	Vanillylmandelic acid	199.16870	12.35	+	11.80	C_9_H_10_O_5_	↑^###^	↑	↓^*∗∗*^	Tyrosine metabolism
9	Catechol	111.01985	9.46	+	13.07	C_6_H_6_O_2_	↑^#^	↓^*∗*^	↓^*∗∗*^	Tyrosine metabolism
10	Linoleate	281.24722	5.86	+	14.84	C_18_H_32_O_2_	↑^##^	↓^*∗*^	↓	Linoleic acid metabolism

↑: the compound was upregulated; ↓: the compound was downregulated.

^#^ *P* < 0.05, compared with control group; ^##^*P* < 0.01, compared with control group; ^###^*P* < 0.001, compared with control group.

^*∗*^ *P* < 0.05, compared with model group; ^*∗∗*^*P* < 0.01, compared with model group; ^*∗∗∗*^*P* < 0.001, compared with model group.

**Table 3:** List of the identification of potential biomarkers among groups.

No.	Biomarker identification	m/z	ppm	ESI mode	Rt/min	Formula	B vs. A	D1 vs.B	D2 vs. D1	Pathway
1	UMP	324.21578	7.38	+	0.78	C_9_H_13_N_2_O_9_P	↓^#^	↓	↑	Pyrimidine metabolism
2	L-Lysine	147.11222	0.07	+	0.98	C_6_H_14_N_2_O_2_	↑	↑^*∗*^	↓^△^	Lysine biosynthesis
3	Carnitine	162.11193	6.44	+	1.13	C_7_H_15_NO_3_	↓	↓^*∗∗*^	↑^△^	Bile secretion
4	L-Arginine	175.11846	1.68	+	1.14	C_6_H_14_N_4_O_2_	↑	↑^*∗*^	↓^△^	Arginine and proline metabolism
5	N-Formylanthranilic acid	166.04688	18.18	+	2.83	C_8_H_7_NO_3_	↓	↓^*∗*^	↓	Tryptophan metabolism
6	Adenosine	265.94792	7.65	−	9.60	C_10_H_13_N_5_O_4_	↓	↓^*∗*^	↑	Purine metabolism
7	Deoxycholic acid	391.28221	0.28	−	12.10	C_24_H_40_O_4_	↑	↑^*∗∗*^	↑	Bile secretion

↑: the compound was upregulated; ↓: the compound was downregulated.

^#^ *P* < 0.05, compared with control group; ^##^*P* < 0.01, compared with control group; ^###^*P* < 0.001, compared with control group.

^*∗*^ *P* < 0.05, compared with model group; ^*∗∗*^*P* < 0.01, compared with model group; ^*∗∗∗*^*P* < 0.001, compared with model group.

^△^ *P* < 0.05, compared with D1 group; ^△△^*P* < 0.01, compared with D1 group; ^△△△^*P* < 0.001, compared with D1 group.

## Data Availability

The data used to support the findings of this study are available from the corresponding author upon request.

## Abbreviations

AIA: Adjuvant-induced arthritis
BS: Paeoniae Radix Alba
CFA: Complete Freund's adjuvant
FZ: Aconiti Lateralis Radix Praeparata
PCA: Principal component analysis
PLS-DA: Partial least squares-discriminant analysis
RA: Rheumatoid arthritis
TCA: Tricarboxylic acid
TIC: Total ion chromatogram
UMP: Uridine 5'-monophosphate.

## Supplementary Materials

Supplementary Materials Supplementary Table S1: the detailed information of each group. Supplementary Table S2: the nutrient composition of rat food (%). Supplementary Figure S1: the PCA score plot of therapeutic dose groups (A vs. B vs. C1 vs. C2) in ESI+ and ESI−. Supplementary Figure S2: the PCA score plot of toxic dose groups (A vs. B vs. D1 vs. D2) in ESI+ and ESI−. Supplementary Figure S3: the mass spectra of the compounds listed in the manuscript.
