# Supplementary material for: Investigation of Efficacy Enhancing and Toxicity Reducing Mechanism of Combination of Aconiti Lateralis Radix Praeparata and Paeoniae Radix Alba in Adjuvant-Induced Arthritis Rats by Metabolomics
Source: Evid Based Complement Alternat Med. 2019 Mar 18;2019:9864841. doi: 10.1155/2019/9864841 (PMC6442482; doi:10.1155/2019/9864841)
Supplement: Supplementary Materials — Supplementary Table S1: the detailed information of each group. Supplementary Table S2: the nutrient composition of rat food (%). Supplementary Figure S1: the PCA score plot of therapeutic dose groups (A vs. B vs. C1 vs. C2) in ESI+ and ESI−. Supplementary Figure S2: the PCA score plot of toxic dose groups (A vs. B vs. D1 vs. D2) in ESI+ and ESI−. Supplementary Figure S3: the mass spectra of the compounds listed in the manuscript. [file 9864841.f1.docx]

**1. The Detail Information of Each Group**

The detail information of each group was shown in **Table S1**. The rat food was purchased from Sichuan DOSSY Laboratory Animal Co., Ltd. (Chengdu, China). The nutrient composition of rat food was shown in **Table S2**.

**TABLE S1:** The detail information of each group.

| Group | Number of animal | Animal model | Diet |
| --- | --- | --- | --- |
| A group (control) | 10 | Normal rats | Distilled water and rat food |
| B group (model) | 10 | AIA rats | Distilled water and rat food |
| C1 group | 10 | AIA rats | Distilled water and rat food |
| C2 group | 10 | AIA rats | Distilled water and rat food |
| D1 group | 10 | AIA rats | Distilled water and rat food |
| D2 group | 8 | AIA rats | Distilled water and rat food |
| Dexamethasone group | 10 | AIA rats | Distilled water and rat food |

**TABLE S2:** The nutrient composition of rat food (%).

| Nutrient composition (%) | Water content ≤ | Crude protein ≥ | Crude fat  ≥ | Crude fiber  ≤ | Ash content ≤ | Calcium | Phosphorus | Lysine ≥ | Methionine+cystine  ≥ |
| --- | --- | --- | --- | --- | --- | --- | --- | --- | --- |
| Rat food | 10 | 18 | 4 | 5 | 8 | 1.0-1.8 | 0.6-1.2 | 1.32 | 0.78 |

**2.** **Principal Component Analysis (PCA)** **of The Manuscript**

The PCA parameter of therapeutic dose groups (positive ion mode: R^2^X=0.482; negative ion mode: R^2^X=0.533) and toxic dose groups (positive ion mode: R^2^X=0.469; negative ion mode: R^2^X=0.496). The PCA score plots in positive and negative modes were shown in **Figure S1, S2**. As the pictures shown, the control group, model group and other administration groups showed an obvious trend of separation. The results suggested that there were great differences in metabolites among all groups. Therefore, further PLS-DA analysis was necessary to study the relationship between the different metabolites of each group.


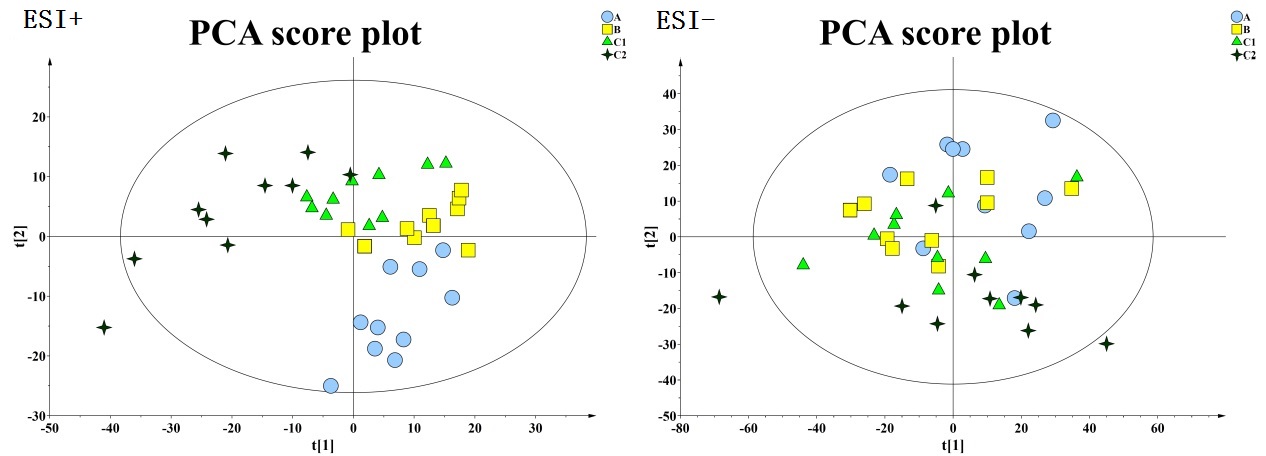


**FIGURE S1**: The PCA score plot of therapeutic dose groups (A vs B vs C1 vs C2) in ESI+ and ESI−. Healthy control group (A). AIA model group (B). Therapy group (C1). Efficacy enhancing combination group (C2).


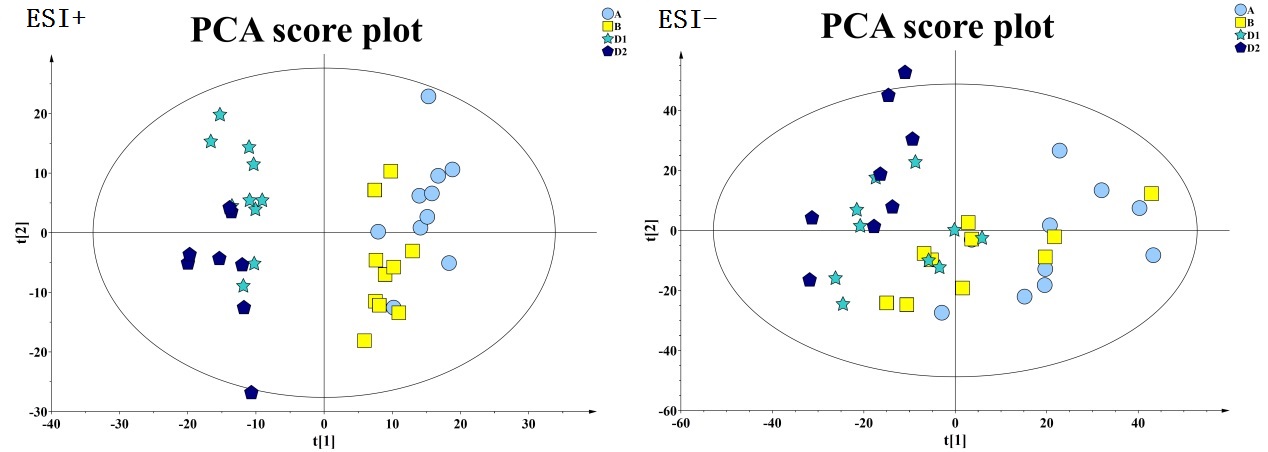


**FIGURE S2**: The PCA score plot of toxic dose groups (A vs B vs D1 vs D2) in ESI+ and ESI−. Healthy control group (A). AIA model group (B). Toxicity group (D1). Toxicity reducing combination group (D2).

**3. The Mass Spectra of The Compounds Listed in The Manuscript**

The mass spectra of the identified compounds were shown in **Figure S3**.


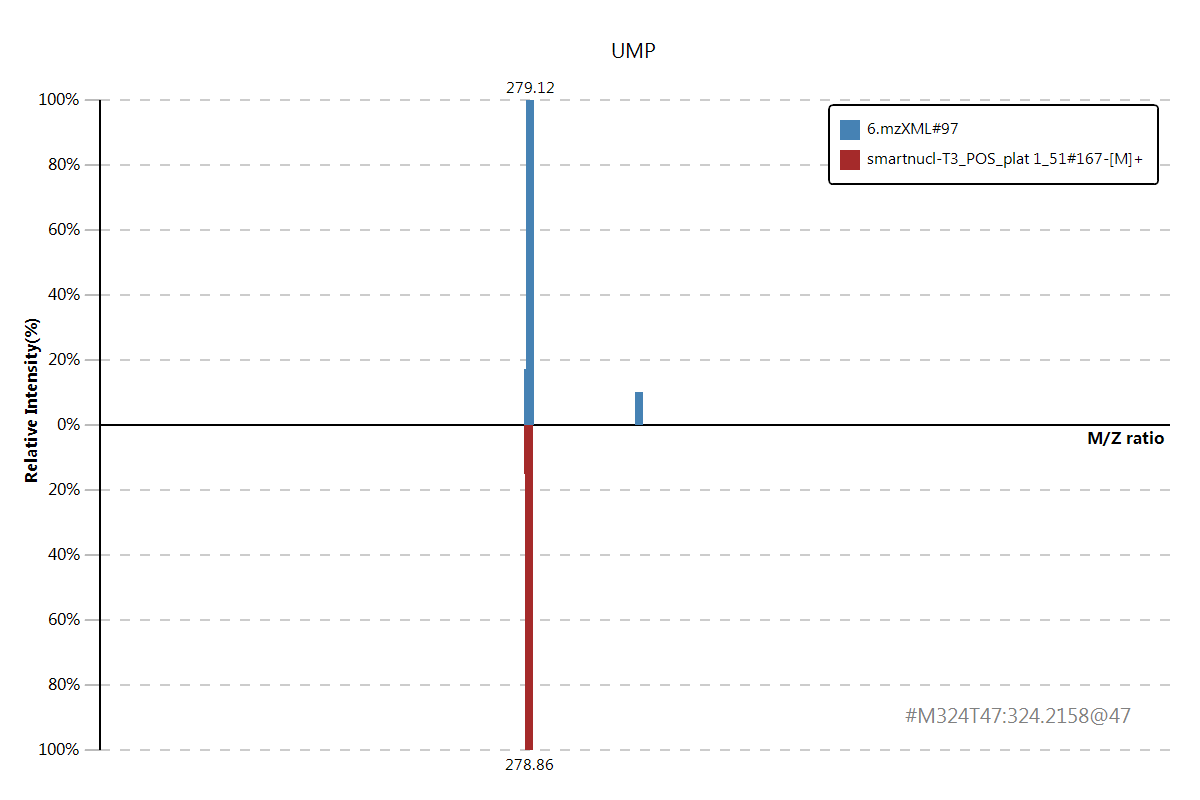

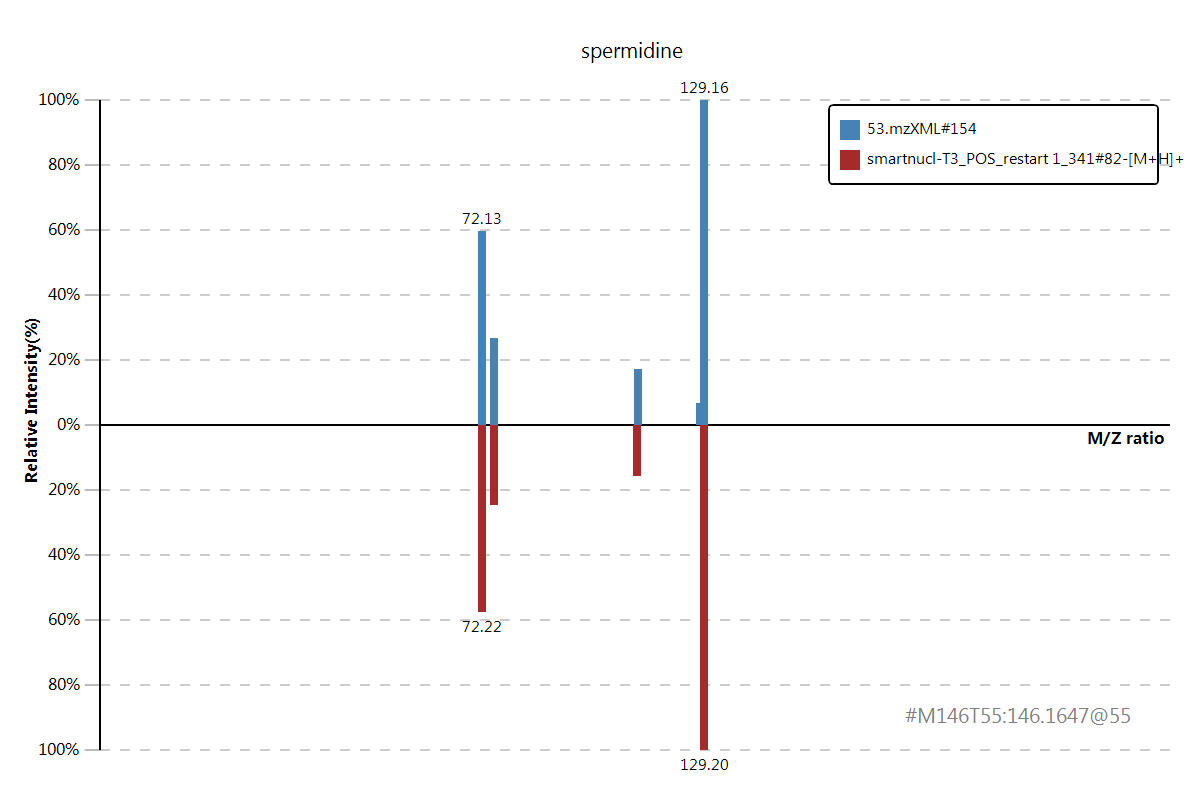

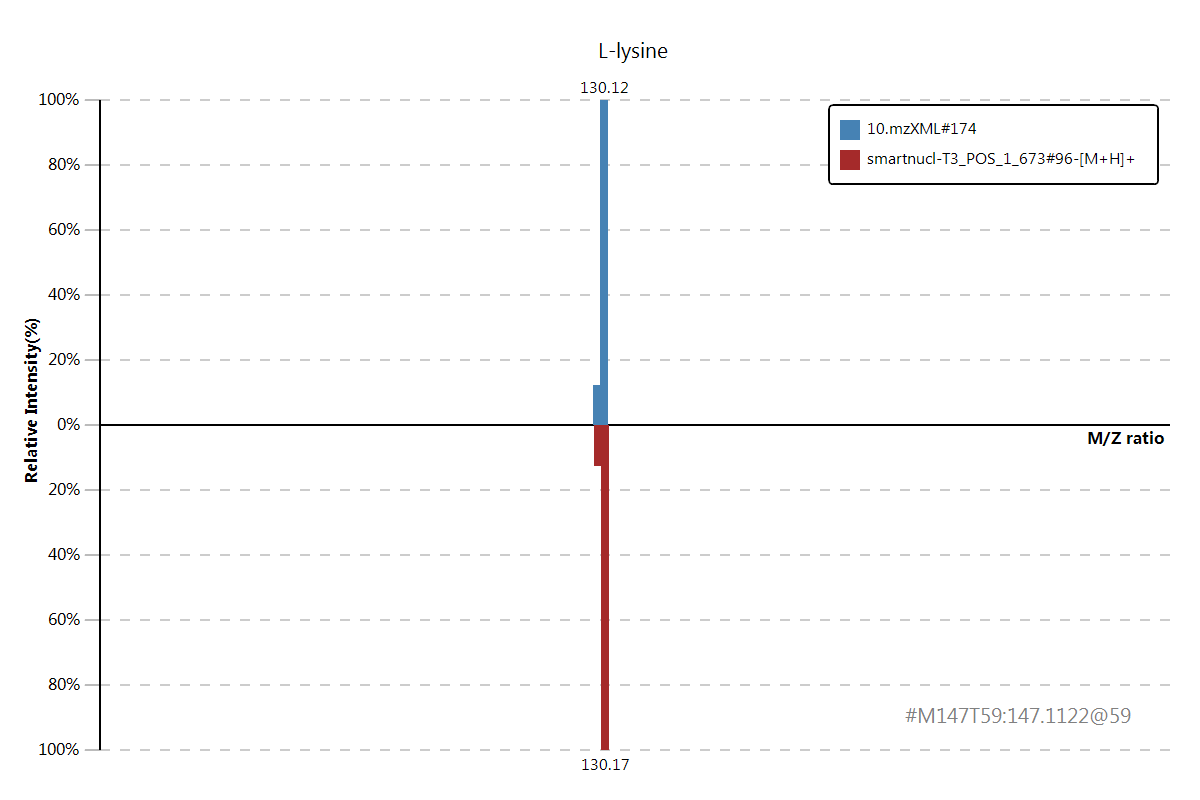

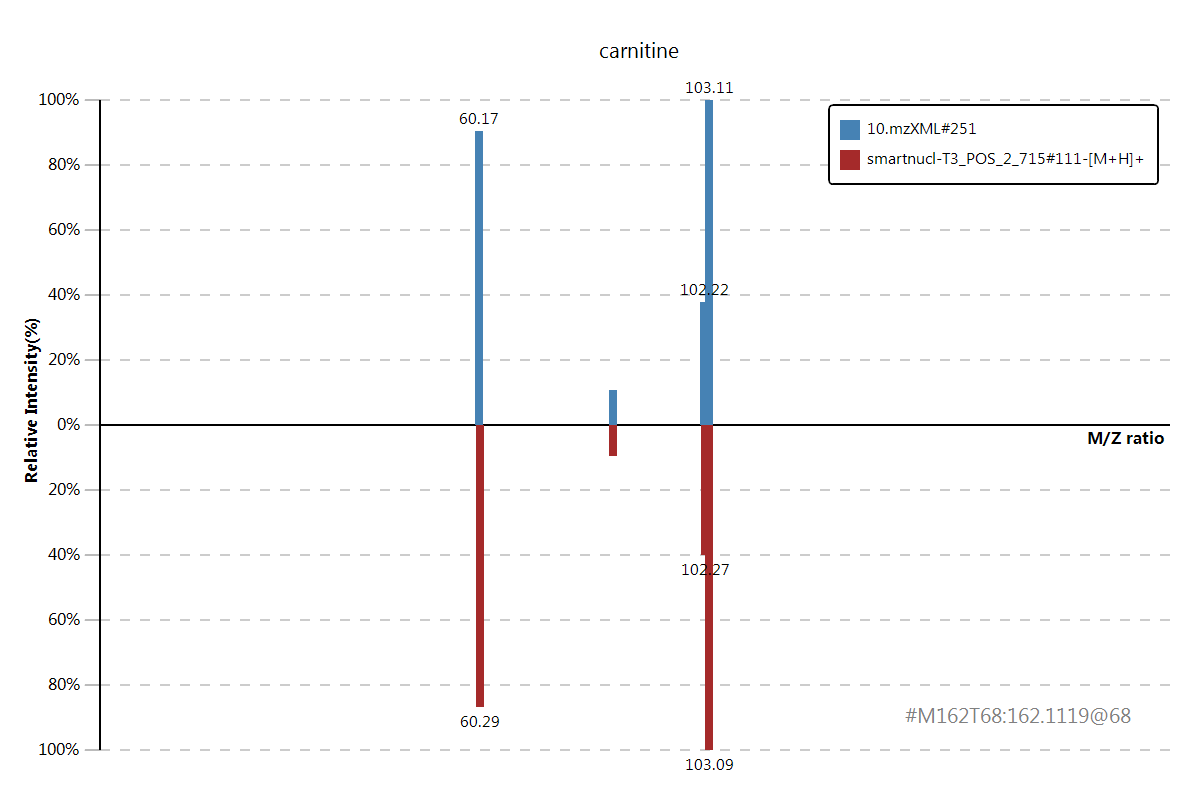

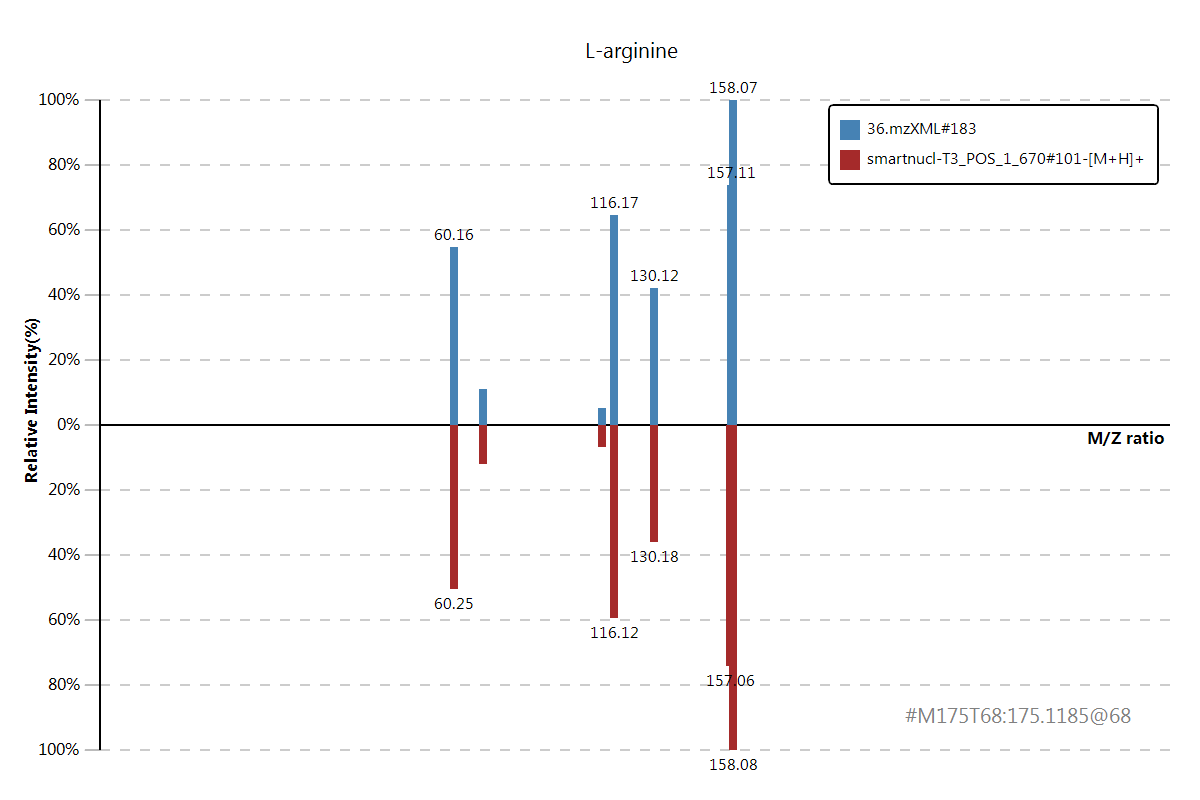

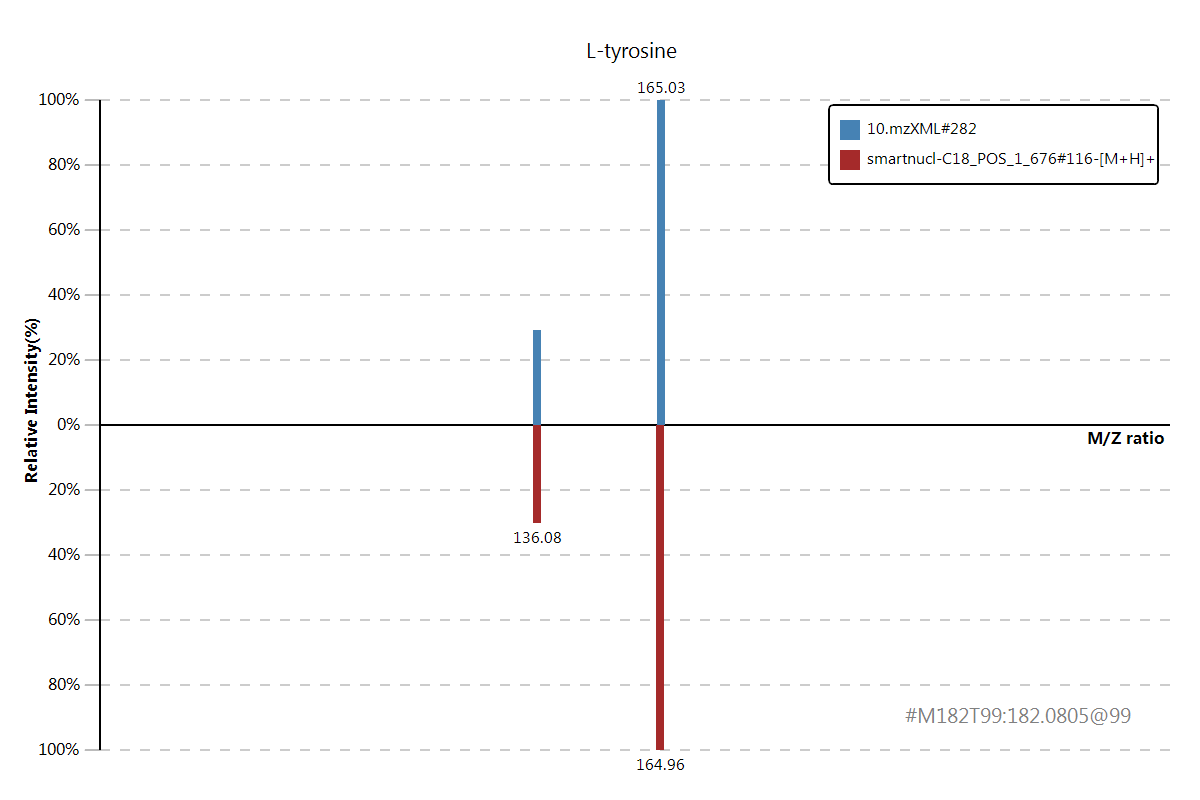

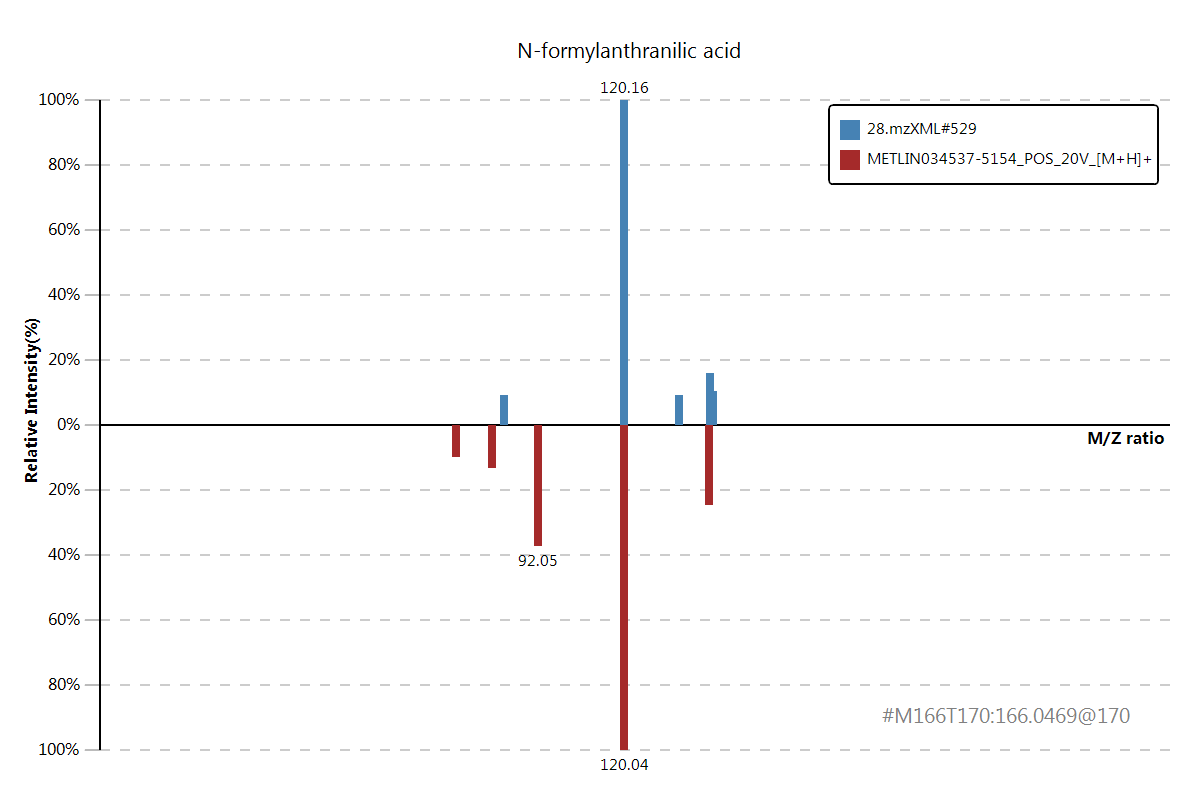

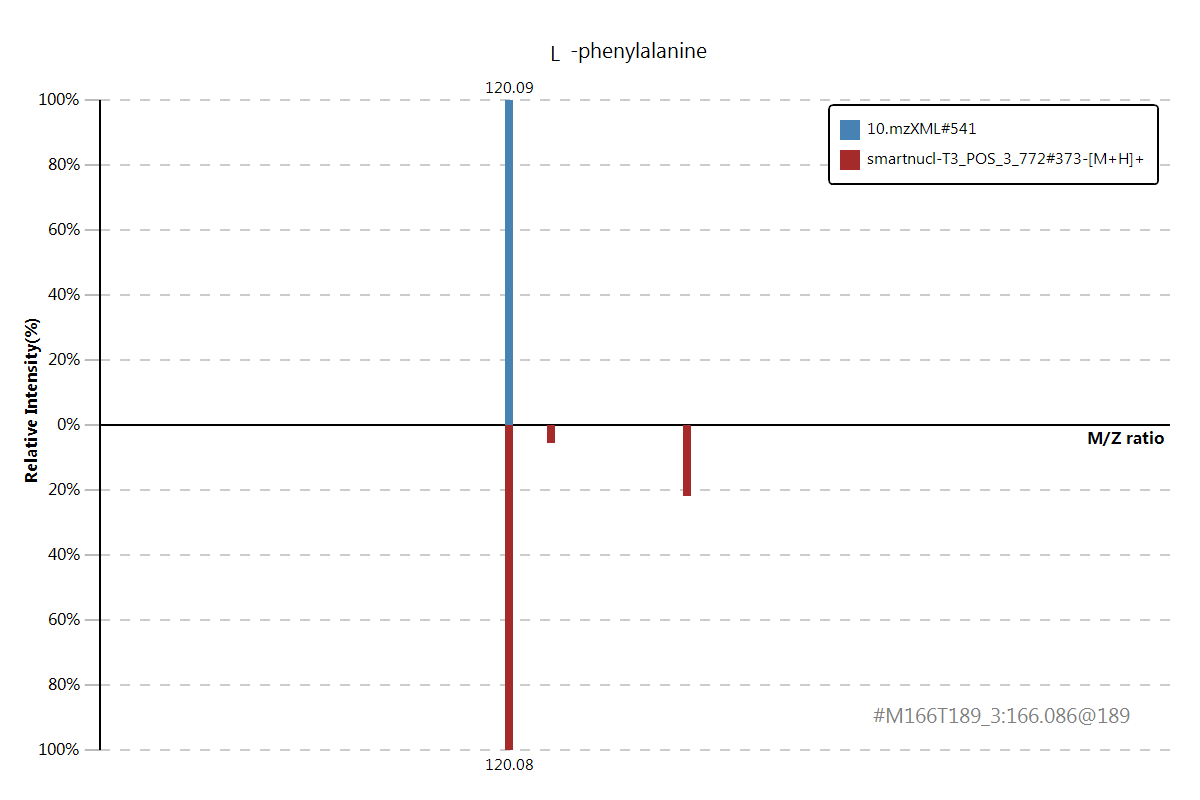

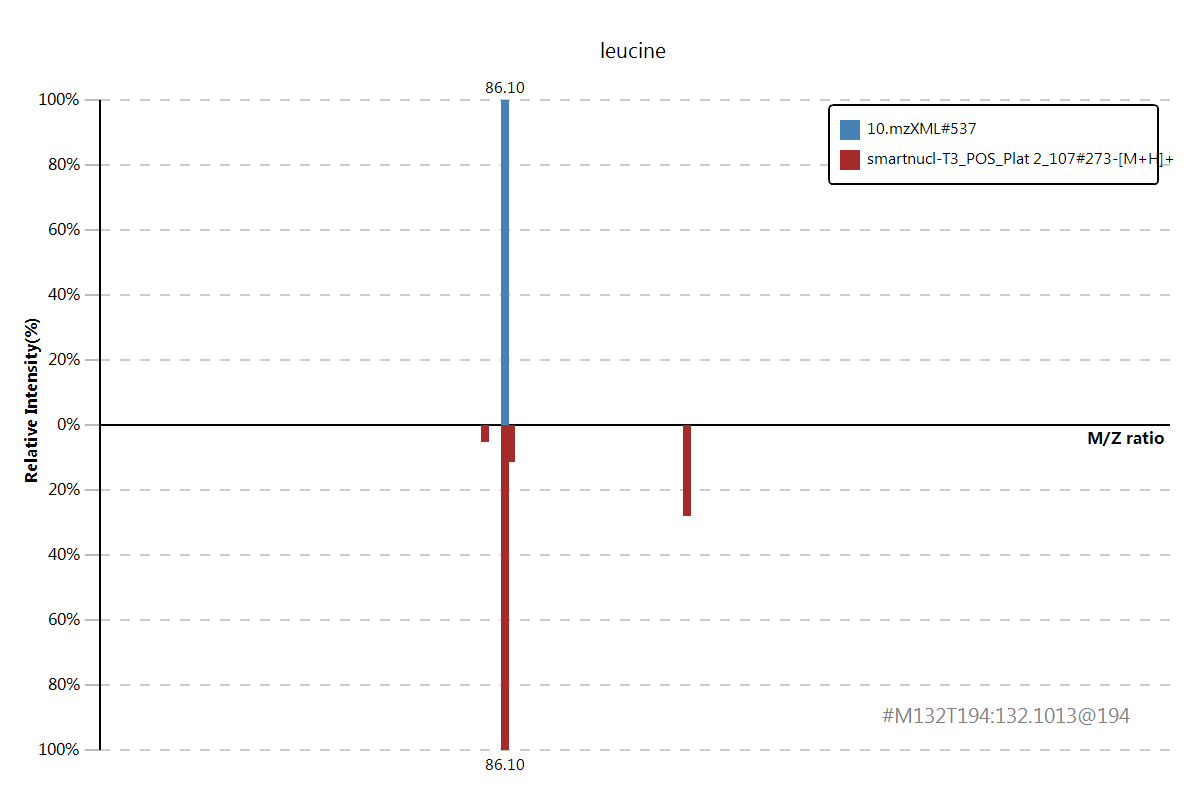

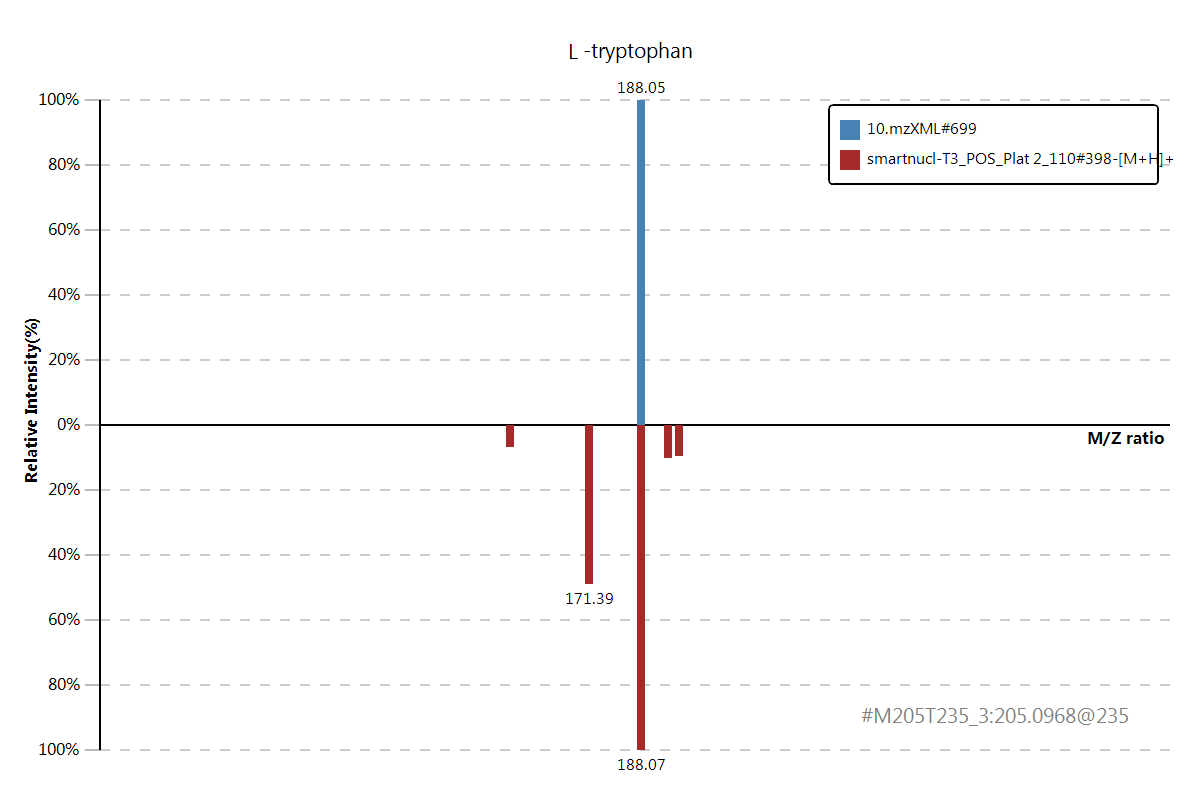

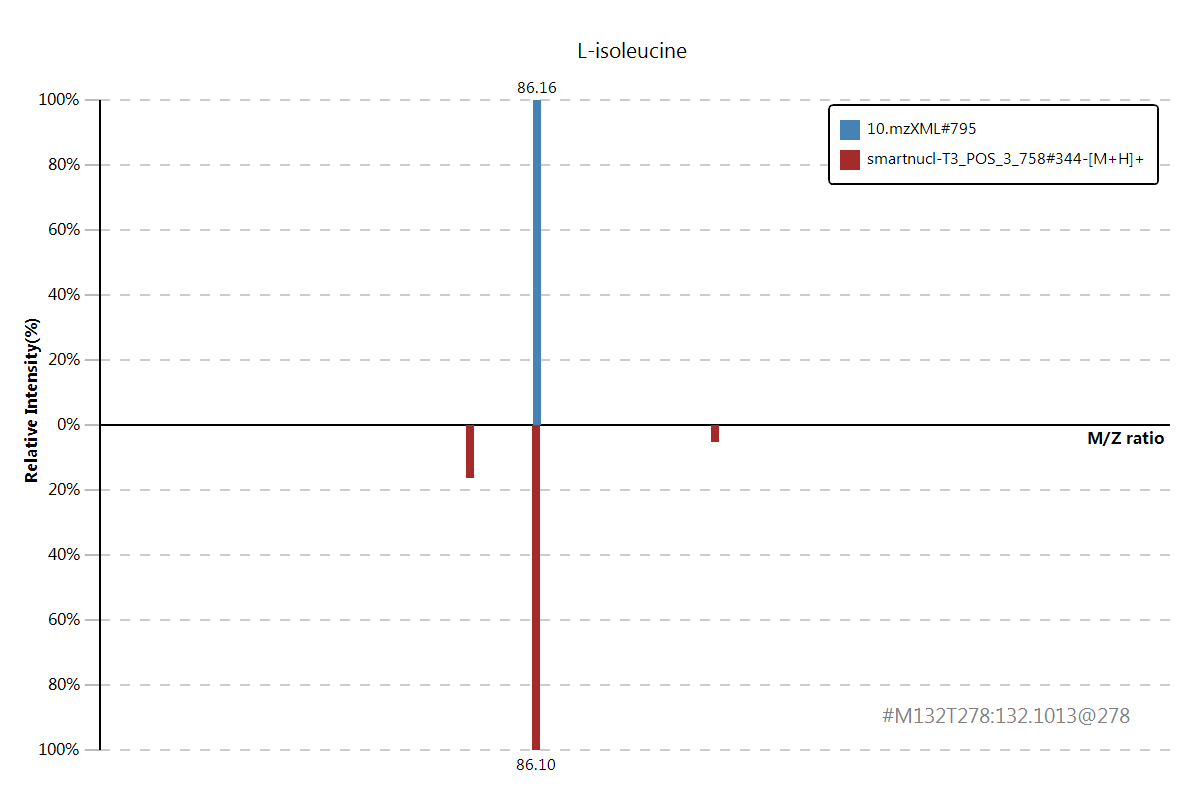

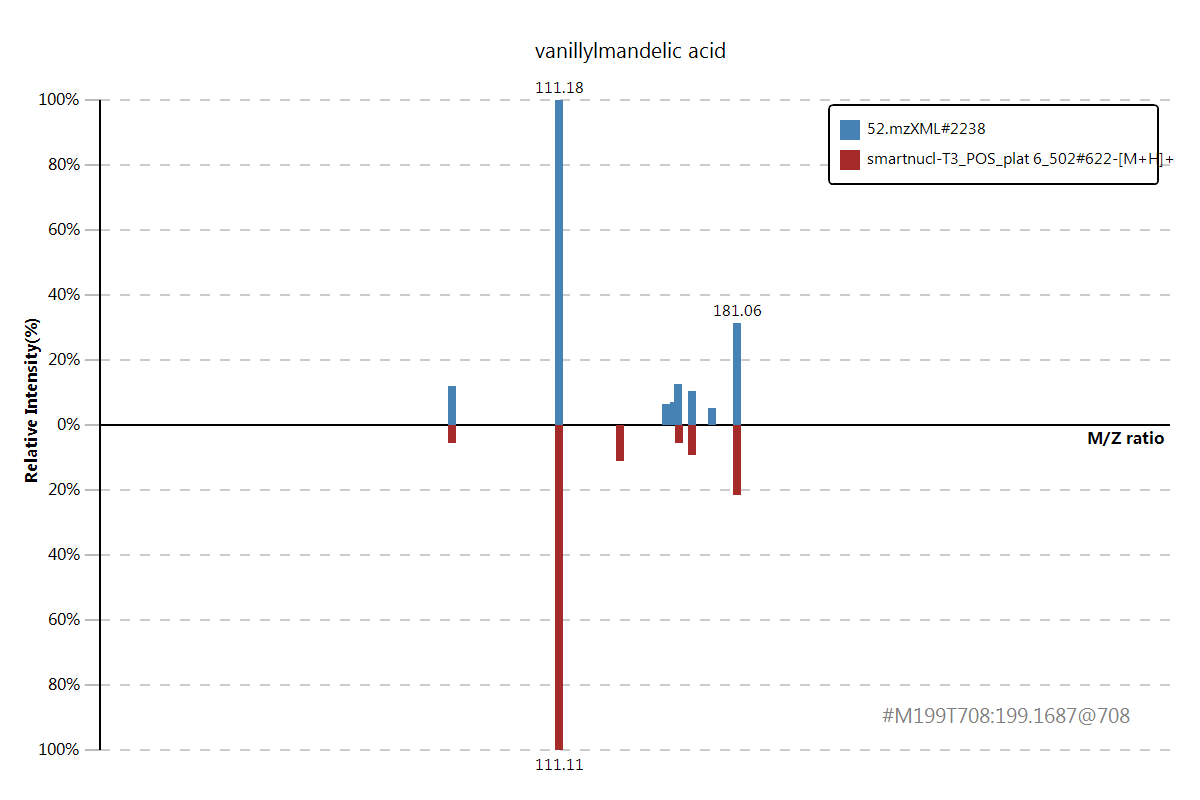

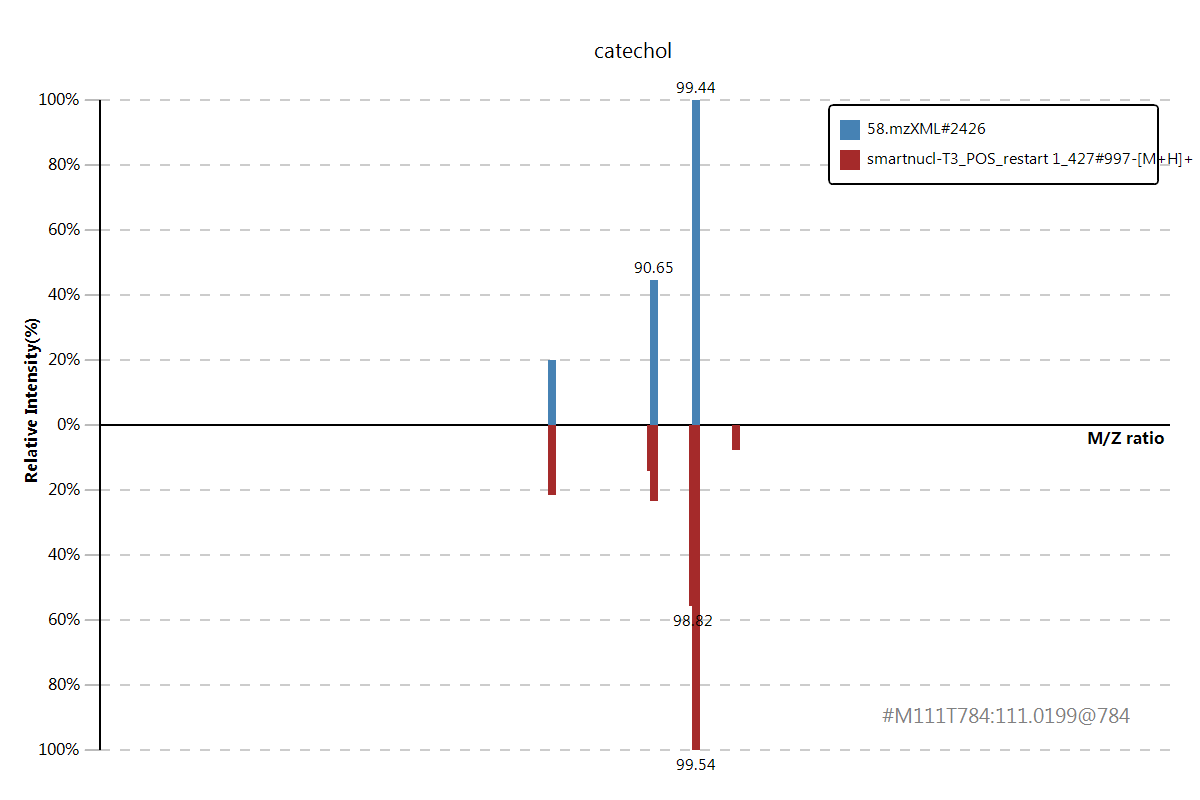

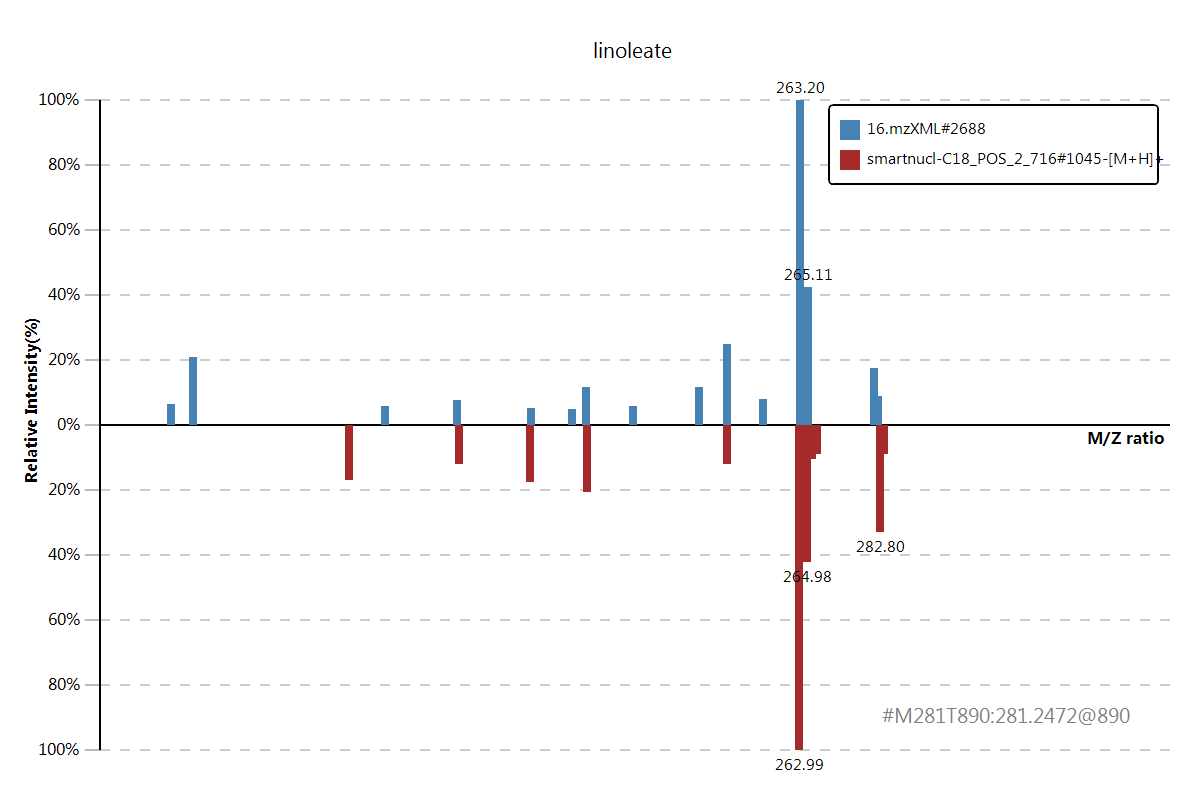

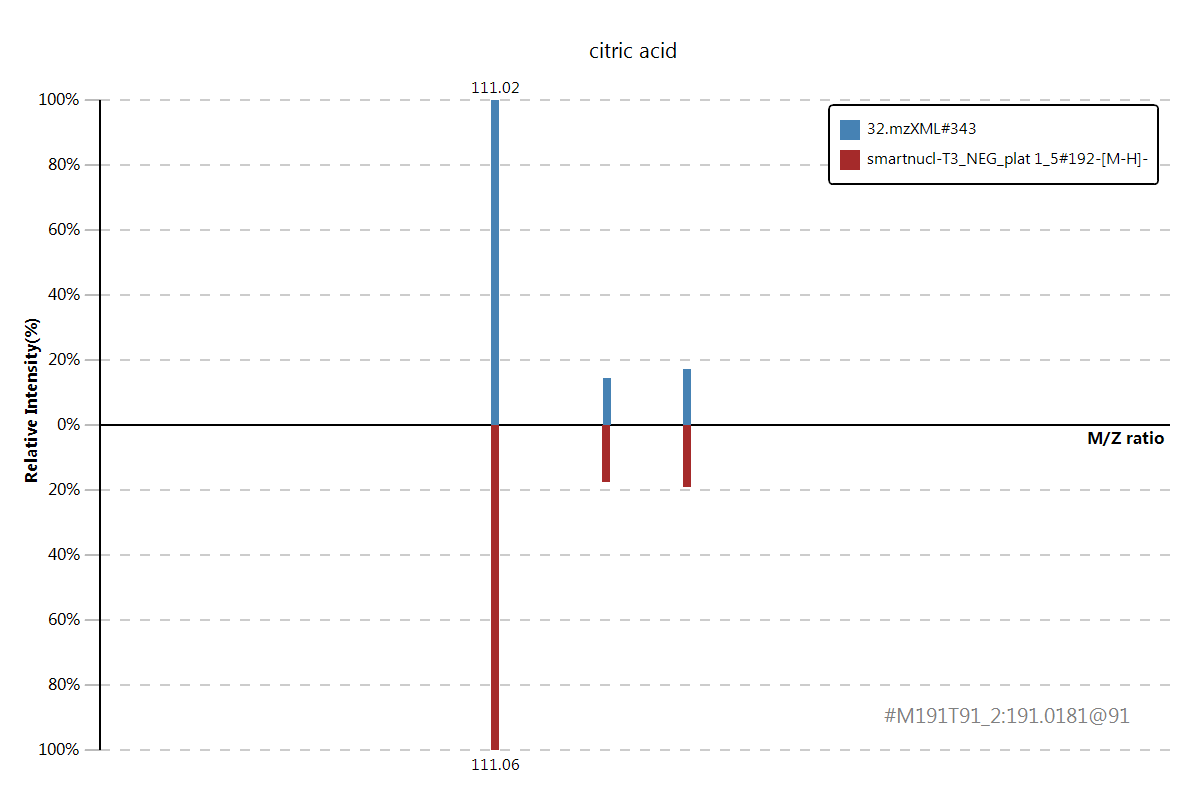

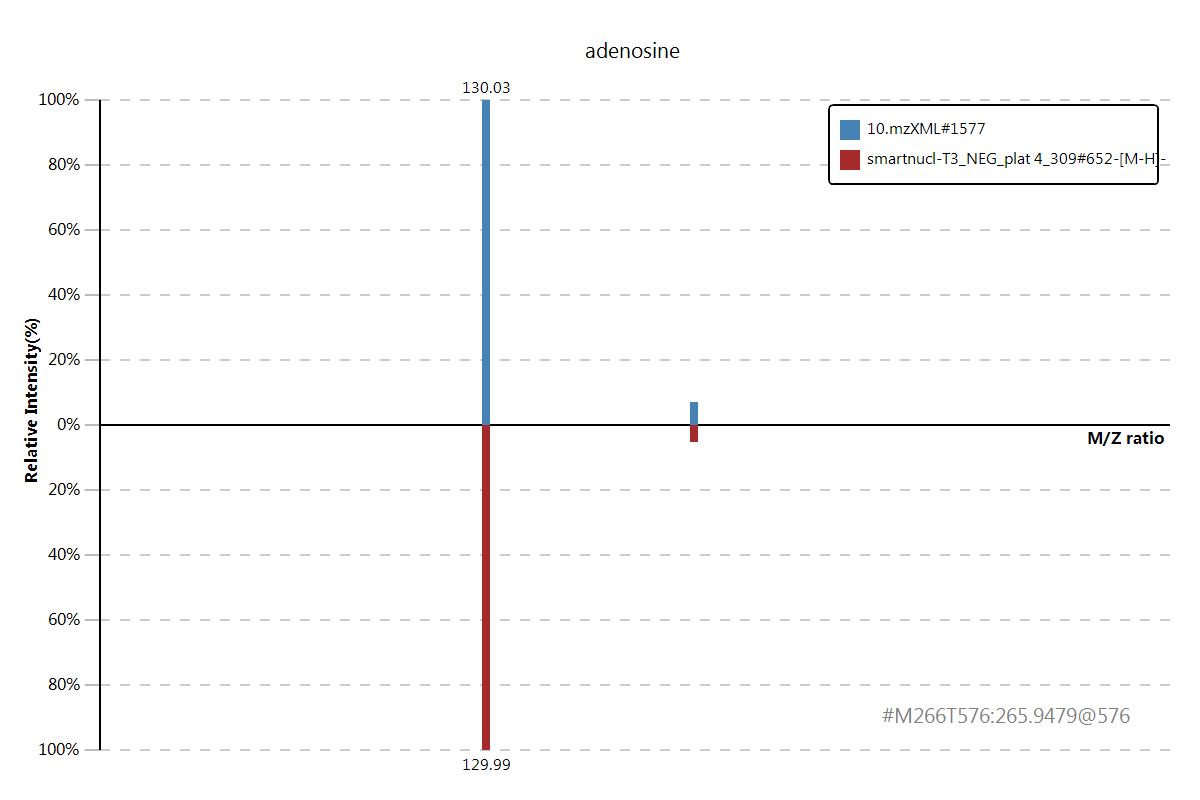

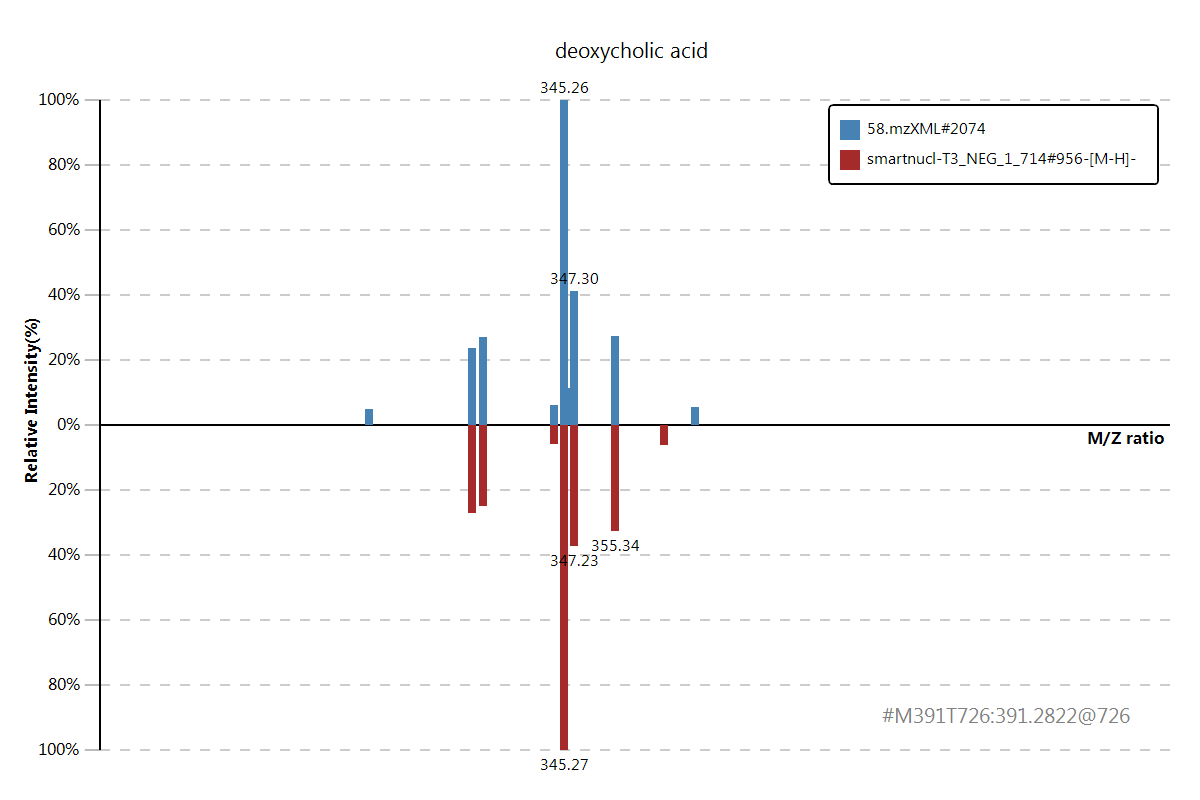


**FIGURE S3:** The mass spectra of the compounds listed in the manuscript.
